# Evaluation of Clustering and Genotype Distribution for Replication in Genome Wide Association Studies: The Age-Related Eye Disease Study

**DOI:** 10.1371/journal.pone.0003813

**Published:** 2008-11-26

**Authors:** Albert O. Edwards, Brooke L. Fridley, Katherine M. James, Anil S. Sharma, Julie M. Cunningham, Nirubol Tosakulwong

**Affiliations:** 1 Department of Ophthalmology, Mayo Clinic, Rochester, Minnesota, United States of America; 2 Division of Biostatistics, Mayo Clinic, Rochester, Minnesota, United States of America; 3 Laboratory Medicine and Pathology, Mayo Clinic, Rochester, Minnesota, United States of America; Ecole Normale Supérieure de Lyon, France

## Abstract

Genome-wide association studies (GWASs) assess correlation between traits and DNA sequence variation using large numbers of genetic variants such as single nucleotide polymorphisms (SNPs) distributed across the genome. A GWAS produces many trait-SNP associations with low p-values, but few are replicated in subsequent studies. We sought to determine if characteristics of the genomic loci associated with a trait could be used to identify initial associations with a higher chance of replication in a second cohort. Data from the age-related eye disease study (AREDS) of 100,000 SNPs on 395 subjects with and 198 without age-related macular degeneration (AMD) were employed. Loci highly associated with AMD were characterized based on the distribution of genotypes, level of significance, and clustering of adjacent SNPs also associated with AMD suggesting linkage disequilibrium or multiple effects. Forty nine loci were highly associated with AMD, including 3 loci (*CFH*, *C2/BF*, *LOC387715/HTRA1*) already known to contain important genetic risks for AMD. One additional locus (*C3*) reported during the course of this study was identified and replicated in an additional study group. Tag-SNPs and haplotypes for each locus were evaluated for association with AMD in additional cohorts to account for population differences between discovery and replication subjects, but no additional clearly significant associations were identified. Relying on a significant genotype tests using a log-additive model would have excluded 57% of the non-replicated and none of the replicated loci, while use of other SNP features and clustering might have missed true associations.

## Introduction

Genetic variation altering the risk of developing common and complex traits or diseases is being discovered using genome wide association studies (GWASs) [1]. Genetic association studies typically seek to identify common sequence variation indirectly associated with a trait. Replication, detailed genotyping, and functional studies subsequently determine which of the variations in a given locus are most likely to be directly associated with disease and which are inherited along with other variants in the population (linkage disequilibrium).

Although a GWAS is a powerful means for discovering trait-associated variants [2], a large number of variants are typically identified with small p-values suggestive of possible association. Most of these variants (e.g., single nucleotide polymorphisms, SNPs) are not associated with disease in subsequent replication studies [3], even with well-designed studies using a larger number of subjects. The large number of SNPs (say 100,000 or more) studied means that with a liberal P-value of 0.001, approximately 100 SNPs would be associated with the trait by chance alone. In addition to association by chance alone, other reasons for spurious association include population stratification and genotyping artifacts [4]. Thus, a major challenge in replication of GWASs is how to define a significant association using p-values or other features.

A number of strategies have been proposed to identify the SNPs more likely to be truly associated with the trait [4], [5]. It is generally accepted that methods (e.g., the Bonferroni correction) based on the number of tests (e.g., SNPs) are overly conservative because the tests are not independent as a result of linkage disequilibrium. Because multiple loci in the genome are being sought in a GWAS, estimates incorporating the prior odds of association and the power to detect association have been proposed [6] and others have discussed the advantages of non-parametric tests based on genotype counts or multilocus modeling [7]–[9]. A commonly employed empirical method is the quantile-quantile plot, where the values of the observed test statistics are plotted against the expected observations [5]. Deviation from the expected suggests the range of potentially significant observations.

Generally SNPs without common minor alleles and accurate genotype call rates are excluded from subsequent analysis, because SNPs without these features are more likely to represent artifacts [4]. It is commonly thought that clustering of SNPs associated with the trait within a chromosomal region excludes genotyping error and may identify regions more likely to harbor disease associated variants [4]. Regardless of the strategies used to select SNPs and loci for replication, an essential component of all GWASs is replication in additional, independent cohorts, preferably with larger sample sizes [3].

In this study, we sought first to test the idea that features of individual SNPs associated with disease and clustering of nearby significant SNPs (i.e., support for association from adjacent SNPs) would be useful in selecting loci from genetic association studies for replication [4]. We hypothesized that true disease-associated SNPs would have genotype distributions that fit a log-additive model as well or better than two degree of freedom χ^2^ tests and would be more likely to have nearby SNPs also associated with disease (due to linkage disequilibrium). The log-additive model is less sensitive to changes driven only by differences in heterozygote frequencies between cases and controls and thus is impacted less by this common effect of genotyping error or population stratification [10]. We also explored the possibility that failure to replicate individual SNPs could arise due to differences in the structure of linkage disequilibrium between discovery and replication subject groups as has been suggested by some investigators [11]. To address these questions we attempted to replicate disease-associated SNPs from a recently released and publically available GWAS through dbGaP ((http://view.ncbi.nlm.nih.gov/dbgap-controlled), accession number phs000001.v1.p1.) on subjects with age-related macular degeneration (AMD; OMIM 603075).

AMD is a common trait with well established genetic risks. By the year 2020, three million people in the United States are expected to have advanced AMD that often leads to severe vision loss [12]. AMD is inherited as a complex trait arising from genetic risks, environmental factors such as smoking, lifestyle and body habitus, and diet/nutritional status [13]. Numerous genomic loci have been identified with replicated association with AMD, as recently reviewed by Edwards and Malek [14]. The regulation of complement activation (RCA) locus contains multiple haplotypes altering AMD risk including the haplotype carrying the Y402H variation in complement factor H (*CFH; Gene ID 3075*) [15]–[18]. Protective variants in the complement pathway were subsequently identified in the complement component 2/B factor (*C2/BF; Gene IDs 712/629*) locus [19]. Recently, two reports of association with variation in the complement component 3 (*C3; Gene ID 718*) locus were published [20], [21]. The chromosome 10q26 region, spanning the hypothetical gene *LOC387715* (Gene ID 387715) and the beginning of the *HTRA1* (Gene ID 5654) gene was the second major locus identified [22]–[25]. Thus, there is strong evidence for the involvement of the innate immune system and at least one other pathway in the pathogenesis of AMD.

The age-related eye disease study (AREDS) was a multi-centered clinical trial, which demonstrated the protective effect of antioxidants and zinc on preventing the exudative complications of AMD [26]. Subjects from this study were used in the most powered GWAS on AMD reported to date and is an appropriate dataset for the present investigation, given the clearly established role of heredity for this disease. These data from 100,000 SNPs on 395 AMD cases and 198 controls without AMD were recently deposited into dbGaP. During the preparation of this manuscript a replication study using discordant sib-pairs was reported [27].

Herein, we report that the AREDS GWAS identified the loci already known at the time the data was deposited into dbGaP, namely the CFH, LOC387715/HTRA1, and C2/BF loci. Of the 57 other loci associated with AMD (P<10^−4^), one additional locus (*C3*) reported during the course of this study [20], [21] was replicated in this study based on highly significant association tests and genotyping with independent technologies in multiple cohorts. All replicated loci were highly significant with the log-additive model, but so were many of the non-replicated loci. Support from adjacent SNP arose secondary to linkage disequilibrium in both replicated and non-replicated loci and was not a useful discriminator. Genotyping of tag-SNPs provided support for failed replication, but did not identify any additional clearly replicated loci in this study.

## Materials and Methods

### Subjects

The AREDS subjects genotyped by the AREDS investigators on the Illumina 100,000 SNP platform consisted of 593 individuals (395 AMD cases, 198 controls). These subjects were recorded as non-Hispanic white (97.6%), non-Hispanic black (2%), Hispanic (0%), Asian or pacific islander (0%), or other (0.34%). Access to the raw data from the AREDS trial was provided via the National Eye Institute. Replication of loci identified in the AREDS GWAS on the Mayo subjects used 744 individuals (444 AMD cases, 300 controls without AMD). The use of these subjects was approved by the institutional review board of the Mayo Clinic, written consent was obtained from all subjects and the study was performed in accordance with the Helsinki declaration. Diagnosis was determined by review of fundus photographs as previously described [15]. Briefly, all subjects diagnosed with AMD had large drusen or more advanced findings and controls had 5 or fewer hard drusen without pigment changes or more advanced findings. Replication of tag- or ns-SNPs associated with AMD in the Mayo subjects was performed on DNA samples from AREDS subjects (1,280 cases, 318 controls without AMD). The AREDS DNA samples were provided by the AREDS Operations Committee. Controls included AREDS control categories control, and control questionable groups 1–4, and AMD cases included AMD categories NV AMD, GA, Both, Large Drusen, Large Drusen Questionable groups 1–3, and Questionable advanced AMD.

### Selection of loci from the AREDS dataset for replication

Results of 2×2 allelic tests publically available on dbGaP were used to select SNPs for further study. SNPs with an uncorrected p-value less than 10^−4^ might represent a significant association with disease and this threshold for replication was employed. Individual genotype counts were not available until late in this study due to the time required to gain access to the dataset and thus were not available initially to perform statistical analyses. Therefore, we reviewed the allele association data for all SNPs with p-values less than 0.0001. The allele frequencies of the 100,000 SNPs were reviewed along with the P-values for association with AMD, Hardy-Weinberg equilibrium (HWE), and support from adjacent SNPs. An attempt was made to replicate all SNPs associated with AMD, except those in the artifact groups (see Table 1 for explanation). The definition of a locus for this project is provided in the footnotes to Table 1.

**Table 1:** Number of loci with at least one SNP significantly associated with AMD in the 100,000 SNP genome-wide scan of the AREDS cohort*.

Group	Hardy-Weinberg equilibrium (HWE) in controls (P≥0.01)	Minor allele frequency>1% in cases or controls	One or more adjacent SNPs associated with AMD**	Number of loci***	Total SNPs with P<10^−4^
Confirmed	Yes	Both	Yes	20	30
Confirmed-Rare	Yes	Either	Yes	6	8
Valid	Yes	Both	No	14	14
Valid-Rare	Yes	Either	No	6	6
**Sub-total**				**46**	**58** ****
Loci already associated with AMD	Yes	Either	Yes	3	25
Artifact - Possible	No	NA	No	20	23
Artifact - Probable	No	Either or Both	Yes	30	65
**Total**				**99**	**171**

* Significant association was defined as P<10^−4^.

** An adjacent SNP associated with AMD refers to the nearest centromeric and telomeric SNP within 50 kb or less genotyped on the Illumina 100,000 SNP genome-wide scan with a p-value≤0.01 for association with AMD. SNPs meeting this criteria are referred to as “confirmed”.

*** SNPs associated with AMD were arbitrarily defined as being in separate loci if they were located at least 500 kb from each other.

**** One of these 58 SNPs (rs7497988, now called rs3985626) could not be genotyped on the Illumina platform and was not studied. The already known and artifact categories were not genotyped, leaving 57 AREDS SNPs in 46 loci.

### Selection of tag and functional SNPs

Non-synonymous SNPs within 50 kb of a significant SNP in the AREDS study (p-value less than 0.0001) were genotyped. Tag-SNPs were selected by inspecting the genomic region of each replicated locus for genes within 20 kb or within an LD block of any SNP associated with AMD. Caucasian data from HapMap in the genomic region of each gene and 2 kb upstream and downstream were used for selecting tag-SNPs. Illumina genotyping scores were obtained for SNPs within the selected genomic regions. SNPs with a score greater than 0.6 were used for further analyses. After merging the files containing Illumina scores with the list of candidate tag-SNPs generated using ldSelect [28] with minor allele frequency of at least 0.05 and r^2^ of at least 0.8, an algorithm for tag-SNP selection was developed so that a single tag-SNP would be selected for each LD bin. This algorithm was applied using SAS Version 8.02 (Cary, NC). Only those SNPs deemed candidate tag-SNPs by ldSelect with an Illumina score greater than or equal to 0.60 and a MAF of greater than or equal to 0.05 were considered for further selection using this algorithm. Tag-SNPs were ultimately selected based upon a functional ranking system wherein non-synonymous coding SNPs were preferentially selected among the tag-SNP candidates in each LD bin, followed by synonymous coding SNPs, SNPs from 5′ untranslated regions (UTRs), SNPs from 3′ UTRs, SNPs from 5′ flanking UTRs, SNPs from 3′ flanking UTRs, and finally SNPs from intronic regions. If an LD bin contained more than one tag-SNP with the same highest function ranking, the SNP with the highest MAF was selected as the tag for that bin. This algorithm was applied to tag-SNPs from the confirmed, confirmed rare, valid, and valid rare categories (Table 1). All other non-synonymous SNPs with Illumina scores greater than or equal to 0.60 and MAFs greater than or equal to 0.05, as well as the significant SNPs from the AREDS dataset, were added to the list of tag-SNPs for each category. LD bins containing only one SNP (referred to hereafter as a “singleton SNP”) were excluded from further analysis. Using the list of SNPs generated from the algorithm described above, gene coverage maps were then produced using R 2.5.0 to visually assess the degree of coverage provided by the tag-SNPs that had been selected, as well as their proximity to the significant SNPs from the AREDS dataset. For large genes with adequate coverage but more than 10 LD bins, only tag-SNPs with LD bins within 10 kb of a significant AREDS SNP were selected for genotyping. For genes with low overall coverage or with tag-SNPs in low proximity to a significant AREDS SNP, additional SNPs were selected from these genes if they were located 1–2 kb upstream or downstream of a significant AREDS SNP and had Illumina scores and MAFs greater than or equal to 0.6 and 0.05, respectively. A final list of tag-SNPs, non-synonymous SNPs, and significant AREDS SNPs was then compiled and examined to ensure that no two SNPs were within 60 bp of each other. SNPs for which this was true were excluded. The resulting SNPs were genotyped as described below.

### Genotyping

We designed an Illumina GoldenGate™ assay for these 243 SNPs such that ninety three percent of the SNPs had Illumina SNP scores >0.6. Genomic DNA samples (250 ng) were genotyped following the Illumina protocol (Illumina, San Diego, CA). Genotype calls were made using the Genotyping module of the BeadStudio 3 software. Genotype clusters were reviewed using the replicate and heritability information of 16 control CEPH trios to refine clustering. Initial laboratory quality assurance relied on the GenCall score, a quality metric indicating the reliability of called genotypes that is generated by the BeadStudio software. The GenCall_10 refers to the 10th percentile GenCall score in a particular distribution of GenCall scores. For loci, it represents the 10th percentile rank for all GenCall scores for that locus. Samples with GenCall_10 scores below 0.4 and/or call rates below 90% and SNPs with call rates below 90% were failed. Quality control for genotype call was assessed by concordance for the control CEPH trio DNA replicates and the sample replicates within each plate (2 per 96 well plate).

### Statistics

Upon receipt of genotype intensities, all SNPs were validated by reviewing the accuracy of genotype discrimination (clustering) methods. SNPs or subjects with call rates lower than 95% or not in Hardy-Weinberg equilibrium (P<0.001) were excluded from further analyses. Any SNP presented in tables in the main manuscript have HWE p-values of 0.05 or higher in controls, unless pointed out in the text. Single SNP analyses on genotype distributions [10], were performed in SAS version 8 (SAS Institute; Cary, NC) using logistic regression assuming a log-additive model where SNPs were coded as 0, 1, or 2 for the number of minor alleles. Fisher's exact tests were also performed on genotype distributions. Intragenic haplotype tests were completed using the score test with a 3 SNP sliding window approach within each gene, as implemented in haplo.stats [29].

## Results

### Loci associated with AMD in the AREDS subjects

Allelic p-values (2×2 χ^2^) were publically available on dbGaP at the time this study was initiated and these statistical tests were used to select SNPs for replication. Table 1 presents the number of loci with allelic p-values of less than 10^−4^ that were identified. Inspection of the loci suggested that they could be characterized based on clustering (support from adjacent SNPs for association with AMD), Hardy-Weinberg equilibrium (HWE), and minor allele frequency. Forty nine loci were highly associated with AMD and had features suggestive of valid genotyping results (confirmed and valid categories in Table 1). Of these 49 loci, there were 46 not already known to be associated with AMD containing a total of 58 SNPs showing association with AMD (p<10^−4^). One AREDS SNP (rs7497988, now called rs3985626) could not be genotyped on the Illumina platform, leaving 57 AREDS SNPs that were studied. Three tag-SNPs (rs12907196, rs12899318, and rs12442417) were studied in place of rs3895626. The details of these 57 AREDS SNPs are presented in Table S1, including analysis of raw genotype data that became available late during the course of this study. Notably, the three loci already known to be associated with AMD (CFH, *LOC387715/HTRA1*, and *C2/BF*) at the time of the release of the AREDS GWAS were associated with AMD. Loci from the X chromosome were excluded, because the hemizygous males were not analyzed separately from females by dbGaP and the association analysis could not be interpreted from the dbGaP data at the start of the study.

### Replication of AREDS SNPs highly associated with AMD in the Mayo subjects

Replication of these 57 SNPs was attempted using the Illumina platform and a larger group of subjects consisting of 444 subjects with AMD and 300 subjects without AMD, hereafter referred to as the Mayo subjects. Only one (rs2230199; *C3*) of the 57 AREDS SNPs was clearly replicated in the Mayo subjects (Table 2). Four other AREDS SNPs showed a trend toward association with AMD with an additive p-value of less than 0.01 (Table 2). Four SNPs failed on the Illumina platform and the one SNP (rs10920091) with minor allele frequency above 5% was genotyped using Taqman and was not associated with AMD. A summary of the biological features and the statistical tests for genotype distributions for these 57 SNPs in the AREDS and Mayo subjects is presented in Table S2. Genotype counts for all SNPs in this study are provided in Table S3.

**Table 2:** The five AREDS SNPs out of 57 genotyped that were most associated with AMD in the replication study with Mayo subjects.*

Locus Description				Gene	AREDS subjects (N = 593)			Mayo subjects (N = 744)		
Chromosome	SNP	Function	Minor allele frequency (AREDS controls)		2×2 Allelic test p-value	Fisher Genotypic test p-value	HWE p-value (controls)	Log-Additive model Genotypic test p-value	Fisher Genotypic test p-value	HWE p-value (controls)
7	rs2341823	intron	0.138	PLXNA4B	2.0E-05	4.8E-05	0.013	0.04	0.12	0.883
9	rs7867504	synonymous	0.382	SLC28A3	8.9E-05	1.7E-04	0.091	0.08	0.22	0.810
16	rs8056814	5′ near gene	0.133	CTRB2, CTRB1, BCAR1	4.8E-06	2.0E-05	0.133	0.02	0.05	0.827
11	rs174602	intronic	0.292	FADS2, FADS3	3.9E-05	3.6E-04	0.369	0.03	0.1	0.516
19	rs2230199	Non-synonymous	0.175	C3	2.8E-05	7.0E-05	0.331	3.7E-05	1.10E-04	0.097

The nsSNP in *C3* was considered replicated.

* Genotype counts are provided in Table S3.

### Detailed study of tag-SNPs and non-synonymous coding SNPs across the AREDS loci in the Mayo subjects

The AREDS study was a multi-centered clinical trial based in the USA without strict racial or ethnic enrollment criteria [26], raising the concern that population substructure might exist within the study subjects. Because of the concern that individual SNPs might not replicate between the AREDS subjects and the Mayo subjects due to differences in linkage disequilibrium, tag-SNP and functional-SNP approaches were used to look for association in the 46 loci. The tag-SNPs were selected using linkage disequilibrium as detailed in the methods section. An additional 225 tag-SNPs and 18 non-synonymous SNPs (nsSNPs) were genotyped in the Mayo subjects. Three of the four loci (5 tag-SNPs) showed a trend toward association with AMD using log-additive modeling (Table 3).

**Table 3:** Attempted replication of AREDS loci using 225 Tag-SNPs and 18 nsSNPs genotyped on the Mayo subjects.

Chromosome	AREDS locus	Function	No. SNPs studied in locus	No. SNPs p<0.01	SNP	Fisher Genotypic test p-value	Log Additive model Genotypic test p-value	Global haplotype simulated p-value**
1	LOC127602	flanking 3′ UTR	11	1	rs1871570	0.009	0.009	0.079
7	NOD1	UTR	9	1	rs2906766	0.004	0.009	0.42
7	PLXNA4B	intron	15	1	rs11773117	0.002	0.017	0.34
17	METT10D	5′ UTR	17	1	rs4790335	0.003	0.64	0.53

The SNPs (all Tag-SNPs) from the 4 loci that showed possible association with AMD in the Mayo subjects are listed in this table.^*^

* Genotype distributions are provided in Table S3.

** Global haplotype refers to the 3-SNP haplotype score across the entire gene using all SNPs.

### Genotyping of AREDS subjects locally for the tag-SNPs associated with AMD in the Mayo subjects

The 3 loci that were possibly associated with AMD in the Mayo subjects by genotyping of tag-SNPs (Table 3) were studied using DNA samples obtained from the AREDS subjects. Six SNPs were genotyped on 1280 cases and 318 controls from the AREDS trial (Table 4). Note that the 593 AREDS subjects that were genotyped in 100,000 SNP GWAS (unpublished) deposited in dbGaP overlap partially with the 1598 AREDS subjects genotyped in our laboratory. One of these SNPs was marginally associated with AMD (Table 4), but was not in HWE (P = 0.000026). Additional studies are needed to understand if this SNP alters the risk of AMD.

**Table 4:** Results of genotyping 6 SNPs from the 3 loci in Table 3 with possible association with AMD in the Mayo subjects on 1,598 (1,280 cases and 318 controls) AREDS subjects.*

Chromosome	Locus	SNP	Function	Mayo Samples		AREDS Samples (Replication subjects)	
				Fisher Genotypic test p-value	Log Additive model Genotypic test p-value	Fisher Genotypic test p-value	Log Additive Model Genotypic test p-value
1	LOC127602	rs1871570	intron	0.009	0.009	0.61	0.49
1	LOC127602	rs12038394	intron	0.07	0.02	0.93	0.7
7	NOD1	rs2906766	5′ untranslated region	0.004	0.009	0.38	0.23
7	PLXNA4B	rs1499300	intron	0.02	0.01	0.16	0.77
7	PLXNA4B	rs2341823	intron	0.12	0.04	0.005	0.01
7	PLXNA4B	rs11773117	intron	0.002	0.01	0.34	0.52

* Genotype distributions are provided in Table S4. Note that METT10D (Table 3) was not genotyped because the log-additive model did not support true association.

### Variables associated with replication using genotype data from the disease-associated SNP and nearby SNPs (clustering)

The results presented above demonstrate that 4 of 49 AREDS loci associated with AMD in the 100,000 SNP GWAS were clearly associated with AMD by replication in an independent group of subjects. Although the number of already known (3 loci) and replicated loci (1) was not large enough to build a predictive model, we inspected the features of both groups of AREDS SNPs (replicated and non-replicated) for insights that might facilitate efficient selection of SNPs for replication (Table S1 and Table 5). Clustering of SNPs associated with AMD was present in both replicated and non-replicated loci. Indeed, except for the regulation of complement activation locus where an extensive linkage disequilibrium block contains CFH and related genes [15], the clustering ranged from 0–2 for both groups of AREDS SNPs. The clustering arose secondary to linkage disequilibrium in both replicated and non-replicated loci based on data from HapMap (www.hapmap.org). We have found the log-additive model for genotype distribution a useful parameter in identifying SNPs that are not truly associated with disease in recent projects [30]. Nonetheless, 60% of non-replicated SNPs had p-values from a log-additive genetic model (1 degree of freedom trend test) more significantly associated with disease than by analyses assuming no genetic mdoel (2 degree of freedom χ^2^ analysis). Other features such as level of significance (p-values) and minor allele frequency were not useful discriminators in this dataset (Table S1).

**Table 5:** SNP and locus variables for the 56 successfully genotyped loci that were studied for replication of the association with AMD.*

Replication Status	Average												
	Minor Allele Frequency	HWE p-value	SNP Call Rate	dbGaP allelic p-value	General model Fisher p-value	Additive model p-value	Minor allele frequency in controls for adjacent upstream SNP	HWE p-value for adjacent upstream SNP	dbGaP alleleic p-value for adjacent upstm SNP	Minor allele frequency in controls for adjacent downstream SNP	HWE p-value for adjacent downstream SNP	dbGaP p-value for adjacent downstream SNP	Number of significant SNPs within 20kb of AREDS SNP
Not-replicated	0.163	0.326	0.998	3.782E-05	1.500E-04	0.158	0.256	0.542	0.295	0.263	0.520	0.243	0.571
Replicated	0.278	0.257	0.995	7.718E-06	2.120E-05	1.003E-05	0.302	0.479	0.339	0.278	0.288	0.135	2.500

* The individual data points from each locus are presented in Table S1.

## Discussion

We identified 49 loci in the AREDS 100,000 SNP GWAS that were associated with AMD (P<10^−4^). Three of these 49 loci were already known to be associated with AMD and the SNPs meeting our replication criteria (Table 1) from the remaining 46 loci were genotyped in a second, independent group of subjects with and without AMD from the Mayo Clinic. One SNP from these loci in the *C3* gene showed association with AMD, while the remaining 56 SNPs did not show clear evidence for association. The SNP in *C3* (rs2230199) has been replicated recently in other studies, while this project was ongoing [20], [21].

The observation that *C3* coding polymorphisms are highly associated with AMD in multiple groups of subjects provides further support for the involvement of the alternative pathway of complement in the pathogenesis of AMD. The Arg80Gly (rs223019) polymorphism corresponds to the electrophoretically slow (Arg) and fast (Gly) forms of C3 and may be the causative polymorphism due to the fast forms probable involvement in other diseases including renal transplant survival, Chagas disease cardiomyopathy, and type II mesangiocapillary glomerulonephritis [31]–[33]. Variation in *C3* is thus the third complement locus strongly implicated in the risk of AMD, in addition to *CFH* and *C2/BF*.

An additional focus of this study was to better understand how strategies for selecting SNPs in a GWAS for subsequent replication would perform empirically. With the recent advancements of genotyping technology, GWASs are being completed in record numbers. The current dilemma faced by researchers is how to determine which SNPs to follow-up in replication and subsequent functional studies. Very low p-values, compared to the overall distribution of p-values in a GWAS, are commonly used to select SNPs for subsequent replication. This strategy would have captured the *CFH* and *LOC387715/HTRA1* loci which had the two highest p-values in the study, 1.4×10^−11^ and 2.5×10^−7^ respectively. However, the p-values for the *C2/BF* and *C3* loci were similar to p-values for other loci which failed to replicate (Table S1) in the AREDS GWAS. Thus, selection of only extremely significant p-values from small genome-wide association studies may not be an effective strategy for identification of disease loci and may miss less significant but replicable associations.

Another commonly used strategy is to apply a log-additive genetic model (trend test) to the genotype distributions for ranking association with disease [7]. Since only allele-association (χ^2^) p-values were available to us at the start of this project and we based our selection of loci for replication on this metric, we can make post-hoc comparisons to the genotype distributions that later became available. Relying on the log-additive model would have been superior to χ^2^ tests based on allelic (2×2) or genotype (2×3) tests for association. Table 5 shows that the non-replicated SNPs had significantly less significant (higher) p-values (mean P = 0.15±0.36) for the log-additive genotype test than did the replicated associations (mean P = 0.00001±0.000016; *t* test, P = 2.22×10^−13^). For example, reliance on the log-additive genetic model test rather than the 2×2 allelic χ^2^ would have excluded 32/56 of the non-replicated SNPs and none (0/4) of the replicated SNPs/loci at the same level of significance (P<10^−4^).

As noted in the introduction, clustering of SNPs significantly associated with case-control status is thought to provide support for the association. While this study was ongoing, we observed clustering of SNPs associated with AMD in toll-like receptor (TLR) loci [30]. The association with AMD was shown to be spurious in extensive replication studies, demonstrating that (as expected) clustering can arise due to linkage disequilibrium with or without true association with disease [30]. We also observed in the present study that clustering of SNPs was not useful in selection of loci for replication (Table S1). We also are not convinced that clustering protects against genotyping error, because copy number variation could extent across a locus with multiple SNPs associated with disease.

The AREDS subjects came from 11 different clinics representing different regions of the USA [26]. In addition to including subjects of different ethnicities, we expected considerable genetic variation between the different clinical sites. Our replication strategy used tag-SNPs selected based on linkage disequilibrium, because variation in linkage disequibrium between SNPs in different populations is a cause of failed replication in GWASs [11], [28]. In addition to genotyping the 46 AREDS loci themselves, we used tag-SNPs to account for differences in linkage disequilibrium between the AREDS subjects and the Mayo subjects. Even though we have preliminary data showing the existence of population substructure within the AREDS subjects (data not shown), none of the 225 tag-SNPs were clearly associated with AMD. Even though the use of tag-SNPs would allow study of haplotype association, there would seem to be minimal, if any value, to including tag-SNPs during the initial phase of replication of GWAS results given the increased cost of genotyping.

Our experience with the AREDS 100,000 SNP GWAS and other studies suggests some simple guidelines for designing GWASs and replication studies that would improve the ability to efficiently detect disease associated loci [34], [35]. It is generally accepted that performing the initial GWAS using 2 or more cohorts of sufficient size is helpful in identifying loci that might be missed in one study. Selection of SNPs for replication should be based upon genotype distributions between cases and controls using appropriate statistical methods [5], [7] and not on 2×2 tests for allelic association, clustering of SNPs, or other features of the loci except for having a minor allele frequency high enough to enable adequate power in the study. Adjustment for multiple testing can be considered and applied, although it is unclear how such methods would perform in actual practice [36]. The threshold for p-values to select the set of SNPs for replication should not be so high as to exclude truly associated loci of modest effects. To facilitate the use of public databases (e.g., dbGaP) of GWASs, the genotype counts should be publically available. The significance results presented should be based on genotype distributions using both the 1 degree of freedom trend test and 2 degree of freedom tests, rather than allelic tests.

## Supporting Information

Table S1 Features of the 56 of 57 AREDS SNPs highly associated with AMD (P<10E-04) in the AREDS 100,000 SNP genome wide association study that were not replicated are shown in the top section of this table. SNPs from the CFH, BF/C2 and LOC387715/HTRA1 loci already known to be associated with AMD and the C3 locus replicated in this study are shown in the bottom section of this table. Abbreviations: HWE, Hardy-Weinberg equilibrium; SNP, single nucleotide polymorphism; AREDS, age-related eye disease study.
(0.17 MB DOC)

Table S2 Biological information and statistical tests of allele and genotype association with AMD in the AREDS subjects (dbGAP data) and Mayo subjects. The replicated SNP in C3 is highlighted.
(0.17 MB DOC)

Table S3 Genotype distribution for all 300 SNPs genotyped on Mayo samples, which includes 57 AREDS SNPs, 225 tagSNPs, and 18 non-synonymous SNPs.
(1.85 MB DOC)

Table S4 Genotype distribution results for 6 SNPs with significant log-additive p-values in Mayo samples compared to genotype distribution results for the same SNPs genotyped in AREDS samples (replication cohort).
(0.06 MB DOC)
