# Correction: Evaluation of Clustering and Genotype Distribution for Replication in Genome Wide Association Studies: The Age-Related Eye Disease Study

**DOI:** 10.1371/annotation/34c41e76-e7dc-4a84-b1b7-c3f895207d9f

**Published:** 2008-12-08

**Authors:** Albert O. Edwards, Brooke L. Fridley, Katherine M. James, Anil K. Sharma, Julie M. Cunningham, Nirubol Tosakulwong

The fourth author's name appears incorrectly. It should be: Anil K. Sharma. The correct citation should read: Edwards AO, Fridley BL, James KM, Sharma AK, Cunningham JM, et al. (2008) Evaluation of Clustering and Genotype Distribution for Replication in Genome Wide Association Studies: The Age-Related Eye Disease Study. PLoS ONE 3(11): e3813. doi:10.1371/journal.pone.0003813

