# Supplementary material for: Evaluation of Clustering and Genotype Distribution for Replication in Genome Wide Association Studies: The Age-Related Eye Disease Study
Source: PLoS One. 2008 Nov 26;3(11):e3813. doi: 10.1371/journal.pone.0003813 (PMC2583911; doi:10.1371/journal.pone.0003813)
Supplement: Table S1 — Features of the 56 of 57 AREDS SNPs highly associated with AMD (P<10E-04) in the AREDS 100,000 SNP genome wide association study that were not replicated are shown in the top section of this table. SNPs from the CFH, BF/C2 and LOC387715/HTRA1 loci already known to be associated with AMD and the C3 locus replicated in this study are shown in the bottom section of this table. Abbreviations: HWE, Hardy-Weinberg equilibrium; SNP, single nucleotide polymorphism; AREDS, age-related eye disease study. (0.17 MB DOC) [file pone.0003813.s001.doc]

| AREDS  SNP | Minor Allele Frequency | HWE p-value | SNP Call Rate | dbGaP allelic p-value | General model Fisher p-value | Additive model p-value | Minor allele frequency in controls for adjacent upstream SNP | HWE p-value for adjacent upstream SNP | dbGaP alleleic p-value for adjacent upstm SNP | Minor allele frequency in controls for adjacent downstream SNP | HWE p-value for adjacent downstream SNP | dbGaP p-value for adjacent downstream SNP | Number of significant SNPs within 20kb of AREDS SNP |
| --- | --- | --- | --- | --- | --- | --- | --- | --- | --- | --- | --- | --- | --- |
| rs10268061 | 0.099 | 0.802 | 1.000 | 2.78E-05 | 4.77E-05 | 2.76E-05 | 0.197 | 1 | 0.697 | 0.441 | 0.772 | 0.483 | 0 |
| rs10920091 | 0.361 | 0.148 | 0.988 | 4.47E-05 | 2.25E-05 | 3.09E-05 | 0.201 | 0.178 | 0.853 | 0.441 | 0.763 | 0.014 | 0 |
| rs11208590 | 0.339 | 0.443 | 1.000 | 1.97E-05 | 5.09E-05 | 1.18E-05 | 0.285 | 1 | 0.031 | 0.182 | 0.363 | 0.291 | 0 |
| rs11620639 | 0.044 | 0.010 | 1.000 | 1.59E-05 | 9.43E-05 | 9.81E-05 | 0.023 | 0.091 | 0.013 | 0.023 | 2.16E-05 | 0.027 | 0 |
| rs11926120 | 0.008 | 0.110 | 1.000 | 1.44E-05 | 4.56E-05 | 0.980377 | 0.288 | 0.599 | 0.627 | 0.005 | 1 | 0.109 | 0 |
| rs12405382 | 0.239 | 0.238 | 1.000 | 5.81E-05 | 0.000368 | 0.000134 | 0.118 | 0.734 | 0.864 | 0.454 | 1 | 0.23 | 1 |
| rs12954274 | 0.342 | 0.785 | 1.000 | 2.49E-05 | 0.000116 | 3.46E-05 | 0.118 | 0.661 | 2.4E-05 | 0.472 | 0.194 | 0.000136 | 1 |
| rs13129209 | 0.339 | 0.771 | 0.997 | 3.93E-06 | 2.58E-05 | 1.21E-05 | 0.368 | 0.877 | 2.1E-07 | 0.369 | 0.356 | 0.406 | 0 |
| rs1367634 | 0.341 | 0.574 | 0.992 | 3.29E-05 | 0.000178 | 4.77E-05 | 0.422 | 0.657 | 4.6E-06 | 0.426 | 0.661 | 2.49E-05 | 2 |
| rs1426054 | 0.483 | 0.951 | 0.997 | 8.10E-05 | 0.000331 | 9.33E-05 | 0.408 | 0.236 | 0.125 | 0.297 | 0.734 | 0.302 | 0 |
| rs1447338 | 0.270 | 0.739 | 0.990 | 2.10E-07 | 1.78E-06 | 7.76E-07 | 0.413 | 1 | 0.668 | 0.433 | 0.883 | 3.93E-06 | 0 |
| rs1508459 | 0.019 | 0.301 | 1.000 | 2.42E-05 | 8.09E-06 | 0.000504 | 0.428 | 1 | 0.361 | 0.358 | 1 | 0.166 | 0 |
| rs1629871 | 0.012 | 0.159 | 0.997 | 6.65E-05 | 0.000181 | 0.001999 | 0.028 | 0.006 | 0.000602 | 0.241 | 0.845 | 0.056 | 1 |
| rs174602 | 0.219 | 0.369 | 1.000 | 3.85E-05 | 0.000363 | 9.53E-05 | 0.348 | 0.752 | 0.392 | 0.421 | 0.661 | 0.226 | 0 |
| rs1781731 | 0.072 | 0.272 | 0.997 | 9.56E-05 | 0.00012 | 0.000103 | 0.441 | 1 | 0.000784 | 0.328 | 0.518 | 0.992 | 1 |
| rs1804644 | 0.033 | 0.184 | 1.000 | 1.27E-05 | 4.98E-05 | 7.13E-05 | 0.387 | 0.651 | 0.244 | 0.318 | 0.741 | 0.11 | 0 |
| rs1968199 | 0.008 | 0.110 | 0.998 | 1.46E-05 | 4.63E-05 | 0.980367 | 0.026 | 0.112 | 0.000112 | 0.018 | 1 | 0.008 | 1 |
| rs2054780 | 0.125 | 0.929 | 1.000 | 6.29E-05 | 0.000137 | 7.16E-05 | 0.026 | 0.004 | 0.000112 | 0.072 | 1 | 0.000136 | 2 |
| rs2055451 | 0.291 | 0.128 | 1.000 | 2.72E-06 | 1.23E-05 | 3.16E-06 | 0.4 | 0.767 | 0.95 | 0.367 | 0.438 | 0.945 | 0 |
| rs2267742 | 0.075 | 0.920 | 1.000 | 4.79E-06 | 7.68E-06 | 4.76E-06 | 0.438 | 0.145 | 0.304 | 0.296 | 0.171 | 0.335 | 0 |
| rs2270951 | 0.445 | 0.187 | 0.998 | 8.19E-05 | 0.000419 | 0.000151 | 0.474 | 0.251 | 0.000114 | 0.472 | 0.197 | 9.41E-05 | 2 |
| rs2297634 | 0.479 | 0.619 | 1.000 | 4.60E-05 | 0.00018 | 5.08E-05 | 0.469 | 0.774 | 0.864 | 0.312 | 0.504 | 0.734 | 0 |
| AREDS  SNP | Minor Allele Frequency | HWE p-value | SNP Call Rate | dbGaP allelic p-value | General model Fisher p-value | Additive model p-value | Minor allele frequency in controls for adjacent upstream SNP | HWE p-value for adjacent upstream SNP | dbGaP alleleic p-value for adjacent upstm SNP | Minor allele frequency in controls for adjacent downstream SNP | HWE p-value for adjacent downstream SNP | dbGaP p-value for adjacent downstream SNP | Number of significant SNPs within 20kb of AREDS SNP |
| rs2341823 | 0.088 | 0.013 | 1.000 | 1.99E-05 | 4.76E-05 | 4.72E-05 | 0.118 | 1 | 0.052 | 0.146 | 0.009 | 0.039 | 0 |
| rs2358259 | 0.008 | 0.110 | 0.998 | 1.39E-05 | 4.42E-05 | 0.980395 | 0.064 | 0.174 | 0.266 | 0.4 | 0.881 | 0.213 | 0 |
| rs3775729 | 0.070 | 0.755 | 0.998 | 3.82E-05 | 8.96E-05 | 5.99E-05 | 0.11 | 1 | 0.000266 | 0.038 | 0.244 | 0.021 | 1 |
| rs3813108 | 0.044 | 0.438 | 1.000 | 5.96E-05 | 0.00015 | 0.000132 | 0.09 | 0.186 | 3.24E-06 | 0.077 | 0.606 | 0.391 | 1 |
| rs4268798 | 0.442 | 0.101 | 1.000 | 6.05E-06 | 9.59E-06 | 4.24E-06 | 0.208 | 0.276 | 8.0E-05 | 0.341 | 0.523 | 7.93E-05 | 1 |
| rs4275419 | 0.011 | 0.159 | 0.988 | 4.23E-05 | 4.33E-05 | 0.003575 | 0.036 | 0.215 | 0.742 | 0.164 | 0.186 | 0.025 | 0 |
| rs435776 | 0.458 | 0.173 | 1.000 | 5.30E-06 | 9.31E-06 | 1.06E-05 | 0.241 | 1 | 0.089 | 0.219 | 0.677 | 0.074 | 2 |
| rs5743371 | 0.045 | 0.010 | 1.000 | 2.94E-05 | 0.000311 | 0.000259 | 0.079 | 0.104 | 3.3E-05 | 0.2 | 0.267 | 0.267 | 1 |
| rs5743373 | 0.043 | 0.078 | 1.000 | 3.29E-05 | 0.000185 | 0.000233 | 0.118 | 0.033 | 0.978 | 0.082 | 0.027 | 2.94E-05 | 1 |
| rs6133075 | 0.038 | 0.275 | 1.000 | 4.50E-05 | 0.000183 | 0.000134 | 0.272 | 1 | 0.738 | 0.255 | 0.191 | 0.335 | 0 |
| rs6137194 | 0.331 | 0.130 | 1.000 | 9.23E-06 | 2.63E-05 | 8.1E-06 | 0.449 | 0.664 | 0.027 | 0.372 | 0.444 | 0.541 | 0 |
| rs6592695 | 0.011 | 0.158 | 1.000 | 3.94E-05 | 4.03E-05 | 0.003469 | 0.49 | 0.567 | 0.031 | 0.235 | 0.84 | 0.847 | 0 |
| rs6691327 | 0.008 | 0.110 | 1.000 | 1.44E-05 | 4.56E-05 | 0.980377 | 0.256 | 0.576 | 0.246 | 0.287 | 0.728 | 0.005 | 0 |
| rs6692452 | 0.008 | 0.110 | 1.000 | 1.44E-05 | 4.56E-05 | 0.980377 | 0.026 | 0.112 | 1.4E-05 | 0.336 | 1 | 0.009 | 1 |
| rs6710260 | 0.156 | 0.126 | 0.998 | 5.77E-05 | 0.000577 | 0.000138 | 0.185 | 0.469 | 0.127 | 0.149 | 0.582 | 0.439 | 0 |
| rs7215857 | 0.148 | 0.236 | 1.000 | 7.95E-05 | 0.000245 | 0.000148 | 0.318 | 0.507 | 0.002 | 0.464 | 0.195 | 6.05E-06 | 2 |
| rs7251282 | 0.008 | 1.000 | 0.995 | 4.42E-05 | 4.56E-05 | 0.982934 | 0.469 | 0.566 | 0.676 | 0.028 | 0.006 | 4.67E-06 | 1 |
| rs7316255 | 0.008 | 0.090 | 0.992 | 4.35E-05 | 0.000138 | 0.981309 | 0.4 | 0.881 | 0.852 | 0.273 | 0.045 | 0.702 | 0 |
| rs7536773 | 0.008 | 0.110 | 1.000 | 1.44E-05 | 4.56E-05 | 0.980377 | 0.021 | 0.071 | 0.000136 | 0.026 | 0.112 | 1.44E-05 | 2 |
| rs7536934 | 0.014 | 0.018 | 1.000 | 3.68E-05 | 0.000322 | 0.001572 | 0.492 | 0.47 | 0.007 | 0.223 | 0.001 | 0.101 | 1 |
| rs7550036 | 0.008 | 0.110 | 1.000 | 1.44E-05 | 4.56E-05 | 0.980377 | 0.305 | 0.504 | 0.543 | 0.021 | 0.071 | 0.000136 | 0 |
| rs7867504 | 0.305 | 0.091 | 1.000 | 8.86E-05 | 0.000166 | 0.000156 | 0.195 | 1 | 0.032 | 0.495 | 0.476 | 0.158 | 0 |
| rs7996685 | 0.067 | 0.625 | 1.000 | 4.94E-05 | 0.000108 | 8.13E-05 | 0.392 | 0.765 | 0.692 | 0.162 | 0.791 | 0.592 | 0 |
| rs8005200 | 0.011 | 0.009 | 1.000 | 1.38E-05 | 0.000136 | 0.006353 | 0.018 | 1 | 0.048 | 0.332 | 0.195 | 0.604 | 0 |
| rs8056814 | 0.081 | 0.133 | 1.000 | 4.82E-06 | 2.02E-05 | 1.19E-05 | 0.272 | 0.588 | 0.019 | 0.151 | 0.779 | 0.025 | 0 |
| AREDS  SNP | Minor Allele Frequency | HWE p-value | SNP Call Rate | dbGaP allelic p-value | General model Fisher p-value | Additive model p-value | Minor allele frequency in controls for adjacent upstream SNP | HWE p-value for adjacent upstream SNP | dbGaP alleleic p-value for adjacent upstm SNP | Minor allele frequency in controls for adjacent downstream SNP | HWE p-value for adjacent downstream SNP | dbGaP p-value for adjacent downstream SNP | Number of significant SNPs within 20kb of AREDS SNP |
| rs8086078 | 0.047 | 0.200 | 1.000 | 3.24E-06 | 2.15E-05 | 1.77E-05 | 0.179 | 1 | 0.074 | 0.079 | 0.34 | 5.96E-05 | 1 |
| rs869224 | 0.410 | 0.119 | 1.000 | 7.26E-05 | 0.000256 | 0.000125 | 0.138 | 0.769 | 0.959 | 0.344 | 0.752 | 0.000755 | 0 |
| rs915196 | 0.016 | 0.031 | 0.990 | 6.30E-06 | 5.44E-05 | 0.0006 | 0.385 | 0.226 | 0.22 | 0.408 | 0.459 | 0.258 | 0 |
| rs9285480 | 0.052 | 0.042 | 1.000 | 6.13E-05 | 0.000297 | 0.000189 | 0.085 | 0.141 | 0.114 | 0.405 | 0.375 | 0.089 | 0 |
| rs9484252 | 0.014 | 0.184 | 0.997 | 9.82E-05 | 0.000253 | 0.001355 | 0.333 | 0.519 | 0.948 | 0.374 | 0.761 | 0.958 | 0 |
| rs9514252 | 0.477 | 0.681 | 0.993 | 6.61E-05 | 0.000374 | 0.000109 | 0.264 | 0.855 | 0.921 | 0.092 | 1 | 0.535 | 0 |
| rs9807370 | 0.446 | 0.213 | 0.998 | 9.41E-05 | 0.000492 | 0.000168 | 0.472 | 0.194 | 8.19E-05 | 0.138 | 0.769 | 0.959 | 2 |
| rs9892878 | 0.267 | 0.460 | 0.998 | 7.93E-05 | 0.000569 | 0.000136 | 0.464 | 0.195 | 6.05E-06 | 0.215 | 0.525 | 0.007 | 2 |
| rs9950970 | 0.341 | 0.748 | 0.985 | 4.56E-05 | 0.000202 | 6.29E-05 | 0.095 | 0.227 | 0.482 | 0.424 | 0.459 | 3.29E-05 | 2 |
| **AVERAGE** | **0.163** | **0.326** | **0.998** | **3.782E-05** | **1.500E-04** | **0.158** | **0.256** | **0.542** | **0.295** | **0.263** | **0.520** | **0.243** | **0.571** |

Loci already known to be associated with AMD (3 loci) at the time project was initiated, and the C3 locus replicated in this study

| rs203674 (CFH) | 0.433 | 0.299 | 0.995 | 1.29E-25 | 1.4E-11 | 1.19E-11 | 0.341 | 0.636 | 2.77E-20 | 0.194 | 0.000865 | 0.044 | 2 |
| --- | --- | --- | --- | --- | --- | --- | --- | --- | --- | --- | --- | --- | --- |
| rs429608 (C2/BF) | 0.108 | 0.092 | 1.000 | 2.63E-06 | 1.5E-6 | 7.13E-06 | 0.408 | 0.554 | 0.882 | 0.241 | 0.242 | 0.005 | 6 |
| rs2014307 (HTRA1) | 0.32 | 0.328 | 1.000 | 4.35E-08 | 2.53E-07 | 1.13E-07 | 0.128 | 0.098 | 0.278 | 0.487 | 0.667 | 0.000347 | 0 |
| rs2230199 (C3) | 0.253 | 0.311 | 0.985 | 2.82E-05 | 6.96E-05 | 3.29E-05 | 0.332 | 0.628 | 0.197 | 0.19 | 0.2422 | 0.489 | 2 |
| **AVERAGE** | **0.278** | **0.257** | **0.995** | **7.718E-06** | **2.120E-05** | **1.003E-05** | **0.302** | **0.479** | **0.339** | **0.278** | **0.288** | **0.135** | **2.500** |
