# Supplementary material for: Evaluation of Clustering and Genotype Distribution for Replication in Genome Wide Association Studies: The Age-Related Eye Disease Study
Source: PLoS One. 2008 Nov 26;3(11):e3813. doi: 10.1371/journal.pone.0003813 (PMC2583911; doi:10.1371/journal.pone.0003813)
Supplement: Table S2 — Biological information and statistical tests of allele and genotype association with AMD in the AREDS subjects (dbGAP data) and Mayo subjects. The replicated SNP in C3 is highlighted. (0.17 MB DOC) [file pone.0003813.s002.doc]

|  |  | SNPs Associated with AMD (p<10E-04) in AREDS loci * | | | | AREDS subjects (N = 593) | | | Mayo Replication Subjects (N = 744) | | |
| --- | --- | --- | --- | --- | --- | --- | --- | --- | --- | --- | --- |
| Category | Chromosome | Most significant SNP in AREDS locus | Position** | Function | Genes in AREDS lLocus | Allelic test  p-value | Fisher genotypic test p-value | HWE  p-value | Additive model genotypic test p-value | Fisher genotypic test p-value | HWE p-value |
| Confirmed | 1 | rs11208590 | 40880736 | intron | RIMS3 | 2.0E-05 | 1.9E-04 | 0.468 | 0.69 | 0.83 | 0.169 |
|  | 1 | rs6692452 | 172021128 | intron | KLHL20, CENPL | 1.4E-05 | 1.8E-05 | 0.127 | 0.59 | 0.68 | 1 |
|  | 1 | rs10920091 | 199241687 | intron | KIF21B | 4.5E-05 | 2.2E-04 | 0.133 | 0.50 | 0.80 | 0.36 |
|  | 1 | rs2054780 | 205288235 | intron | YOD1, C1orf116, PFKFB2 | 6.3E-05 | 5.5E-04 | 0.853 | 0.94 | 0.43 | 0.244 |
|  | 1 | rs435776 | 226561413 | non-synonymous | OBSCN, C1orf145, KIAA1639 | 5.3E-06 | 6.6E-05 | 0.263 | 0.12 | 0.30 | 0.722 |
|  | 4 | rs3775729 | 71921548 | intron | GRSF1 | 3.8E-05 | 7.3E-05 | 0.625 | 0.27 | 0.34 | 0.010 |
|  | 4 | rs1447338 | 181280389 | intragenic | MGC45800 | 2.1E-07 | 7.8E-06 | 0.267 | 0.21 | 0.46 | 0.273 |
|  | 4 | rs13129209 | 181313181 | intragenic | MGC45800 | 3.9E-06 | 7.9E-05 | 0.478 | 0.66 | 0.69 | 0.721 |
|  | 6 | rs1781731 | 38790310 | intragenic | DNAH8, GLO1 | 9.6E-05 | 5.8E-04 | 0.124 | 0.97 | 0.40 | 0.706 |
|  | 6 | rs9285480 | 135661689 | intron | AHI1 | 6. 3E-05 | 2.1E-04 | 0.035 | 0.75 | 0.75 | 1 |
|  | 7 | rs5743373 | 30431486 | 3’ untranslated | NOD1 | 3.3E-05 | 9.0E-04 | 0.029 | 0.95 | 1.00 | 1 |
|  | 7 | rs5743371 | 30431899 | intron | NOD1 | 2.9E-05 | 0.0014 | 0.002 | 0.80 | 0.89 | 1 |
|  | 7 | rs2341823 | 131744403 | intron | PLXNA4B | 2.0E-05 | 1.5E-05 | 0.011 | 0.04 | 0.10 | 0.982 |
|  | 9 | rs7867504 | 86110056 | synonymous | SLC28A3 | 8.9E-05 | 4.5E-05 | 0.087 | 0.03 | 0.096 | 0.488 |
|  | 14 | rs8005200 | 53376151 | intragenic | BMP4 | 1.4E-05 | 5.2E-05 | 0.012 | 0.84 | 1.00 | 1 |
|  | 14 | rs11620639 | 56946018 | intron | NAT12 | 1.6E-05 | 6.9E-05 | 0.081 | 0.93 | 0.57 | 0.482 |
|  | 16 | rs8056814 | 73809828 | 5’ near gene | CTRB2, CTRB1, BCAR1 | 4.8E-06 | 4.3E-06 | 0.151 | 0.02 | 0.06 | 0.785 |
|  | 17 | rs7215857 | 2288100 | intron | METT10D | 8.0E-05 | 3.7E-04 | 0.055 | 0.48 | 0.76 | 0.563 |
|  | 17 | rs4268798 | 2313335 | intron | METT10D | 6.1E-06 | 3.9E-05 | 0.096 | 0.40 | 0.67 | 0.606 |
|  | 17 | rs9892878 | 2324062 | intron | METT10D | 7.9E-05 | 0.0024 | 0.249 | 0.84 | 0.57 | 0.161 |
|  | 18 | rs9950970 | 49117806 | intron | DCC | 4.6E-05 | 3.2E-04 | 0.500 | 0.53 | 0.46 | 0.111 |
|  | 18 | rs1367634 | 49118666 | intron | DCC | 3.3E-05 | 2.3E-04 | 0.394 | 0.49 | 0.50 | 0.134 |
|  | 18 | rs12954274 | 49119872 | intron | DCC | 2.5E-05 | 1.7E-04 | 0.538 | 0.50 | 0.45 | 0.068 |
|  | 18 | rs2270951 | 49196651 | intron | DCC | 8.2E-05 | 3.4E-04 | 0.061 | 0.66 | 0.44 | 0.523 |
|  | 18 | rs9807370 | 49197243 | intron | DCC | 9.4E-05 | 3.6E-04 | 0.061 | 0.69 | 0.47 | 0.523 |
|  | 18 | rs869224 | 49231796 | intron | DCC | 7.3E-05 | 2.1E-04 | 0.056 | 0.86 | 0.26 | 0.150 |
|  | 18 | rs8086078 | 69950556 | intron | FBXO15 | 3.2E-06 | 4.8E-05 | 0.108 | 0.85 | 0.89 | 1 |
|  | 18 | rs3813108 | 69966466 | 5’ near gene | C18orf55 | 6.0E-05 | 3.6E-03 | 0.326 | 0.89 | 0.89 | 1 |
|  | 19 | rs7251282 | 1359889 | intron | DAZAP1, NDUFS7, DAZAP1 | 4.4E-05 | 1.8E-05 | 1.000 | 0.84 | 1.00 | 1 |
|  | 20 | rs6137194 | 20896861 | intragenic | C20orf19 | 9.2E-06 | 1.1E-05 | 0.628 | 0.98 | 0.87 | 0.152 |
| Confirmed-rare | 1 | rs7550036 | 171948988 | 5' near gene | KLHL20, CENPL | 1.4E-05 | 1.8E-05 | 0.127 | 0.18 | 0.31 | 1 |
|  | 1 | rs7536773 | 172002001 | synonymous | KLHL20, CENPL | 1.4E-05 | 1.8E-05 | 0.127 | 0.35 | 0.39 | 1 |
|  | 1 | rs6691327 | 172080071 | intron | DARS2, CENPL | 1.4E-05 | 1.8E-05 | 0.127 | 0.18 | 0.31 | 1 |
|  | 1 | rs7536934 | 205328510 | 5’ near gene | C4BPB, PFKFB2, C4BPA | 3.7E-05 | 1.1E-04 | 0.024 | 0.77 | 1.00 | 1 |
|  | 1 | rs4275419 | 223354099 | intron | LOC127602 | 4.2E-05 | 1.4E-05 | 0.182 | 0.97 | 1.00 | 1 |
|  | 7 | rs1968199 | 102541007 | intron | NAPE-PLD, ARMC10 | 1.5E-05 | 1.8E-05 | 0.127 | 0.64 | 1.00 | 1 |
|  | 11 | rs6592695 | 76409173 | 3’ untranslated | PHCA/B3BNT6 | 3.9E-05 | 1.3E-05 | 0.181 | Failed SNP^1^ | Failed SNP^1^ | Failed SNP^1^ |
|  | 15 | rs1629871 | 43510420 | intron | C15orf48, SPATA5L1 | 6.7E-05 | 5.5E-04 | 0.127 | Failed SNP^2^ | Failed SNP^2^ | Failed SNP^2^ |
| Valid | 1 | rs12405382 | 19421568 | intron | KIAA0090,  ZUBR1 | 5.8E-05 | 0.004 | 0.220 | 0.18 | 0.41 | 0.621 |
|  | 1 | rs2297634 | 94349556 | intron | ABCA4 | 4.6E-05 | 5.7E-04 | 0.229 | 0.97 | 0.97 | 0.955 |
|  | 2 | rs6710260 | 69183622 | intron | ANTXR1 | 5.8E-05 | 2.7E-04 | 0.307 | 0.59 | 0.86 | 0.219 |
|  | 3 | rs2055451 | 79924819 | intragenic |  | 2.7E-06 | 1.2E-05 | 0.029 | 0.18 | 0.11 | 0.022 |
|  | 3 | rs1426054 | 141101347 | intragenic |  | 8.1E-05 | 0.002 | 0.705 | 0.84 | 0.58 | 0.280 |
|  | 7 | rs2267742 | 31107071 | intron | ADCYAP1R1 | 4.8E-06 | 6.1E-06 | 0.979 | 0.47 | 0.64 | 0.817 |
|  | 7 | rs10268061 | 115178762 | intragenic |  | 2.8E-05 | 3.2E-05 | 0.672 | 0.30 | 0.51 | 0.138 |
|  | 11 | rs174602 | 61380990 | intron | FADS2, FADS3 | 3.9E-05 | 5.5E-04 | 0.321 | 0.06 | 0.17 | 0.793 |
|  | 13 | rs9514252 | 103808495 | intragenic |  | 6.6E-05 | 0.004 | 0.919 | 0.18 | 0.30 | 0.173 |
|  | 13 | rs7996685 | 109462930 | intragenic |  | 4.9E-05 | 1.8E-04 | 0.429 | 0.37 | 0.59 | 0.659 |
|  | 19 | rs2230199 | 6669387 | nonsyn | C3/GPR108 | 2.8E-05 | 4.8E-04 | 0.448 | 1.8E-05 | 5.6E-05 | 0.137 |
|  | 20 | rs6133075 | 3995407 | intragenic |  | 4.5E-05 | 2.7E-04 | 0.853 | 0.73 | 0.38 | 0.016 |
|  | 20 | rs1804644 | 43005631 | synon | TOMM34 | 1.3E-05 | 4.7E-04 | 0.072 | 0.28 | 0.45 | 0.296 |
| Valid-Rare | 2 | rs2358259 | 174056289 | intragenic | LOC643997 | 1.4E-05 | 1.7E-05 | 0.127 | 0.66 | 0.65 | 1 |
|  | 3 | rs11926120 | 183258616 | intragenic |  | 1.4E-05 | 1.8E-05 | 0.127 | 0.84 | 1.00 | 1 |
|  | 4 | rs1508459 | 98728003 | intron | MGC46496 | 2.4E-05 | 1.7E-06 | 0.342 | 0.739 | 1.00 | 1 |
|  | 6 | rs9484252 | 139603211 | 3’ untranslated | TXLNB | 9.8E-05 | 8.2E-05 | 0.211 | 0.44 | 0.55 | 1 |
|  | 10 | rs915196 | 99082576 | 3’ untranslated | FRAT2, FRAT1 | 6.3E-06 | 1.5E-05 | 0.041 | Failed SNP^1^ | Failed SNP^1^ | Failed SNP^1^ |
|  | 12 | rs7316255 | 75055956 | intragenic | LOC641695 | 4.4E-05 | 7.0E-04 | 0.061 | 0.92 | 1.00 | 1 |

* AREDS loci refer to a 40kb region surrounding a significant (i.e. P <10E-04) AREDS SNP

**Position based on NCBI Genome Build 36.3

^1^ Call rate = 0

^2^ Call rate = 0.76
