# Supplementary material for: Evaluation of Clustering and Genotype Distribution for Replication in Genome Wide Association Studies: The Age-Related Eye Disease Study
Source: PLoS One. 2008 Nov 26;3(11):e3813. doi: 10.1371/journal.pone.0003813 (PMC2583911; doi:10.1371/journal.pone.0003813)
Supplement: Table S3 — Genotype distribution for all 300 SNPs genotyped on Mayo samples, which includes 57 AREDS SNPs, 225 tagSNPs, and 18 non-synonymous SNPs. (1.85 MB DOC) [file pone.0003813.s003.doc]

| **Chromosome** | **AREDS Locus** | **SNP** | **Genotype (# minor alleles)** | **# AMD** | **# Controls** | **Additive p-value** |
| --- | --- | --- | --- | --- | --- | --- |
| 1 | KIAA0090 | rs12405382 | 0 | 242 | 180 | 0.103 |
|  |  |  | 1 | 174 | 106 |  |
|  |  |  | 2 | 27 | 13 |  |
| 1 | KIAA0090 | rs2073105 | 0 | 236 | 179 | 0.103 |
|  |  |  | 1 | 174 | 99 |  |
|  |  |  | 2 | 30 | 18 |  |
| 1 | KIAA0090 | rs2275403 | 0 | 143 | 95 | 0.859 |
|  |  |  | 1 | 208 | 147 |  |
|  |  |  | 2 | 91 | 57 |  |
| 1 | KIAA0090 | rs710882 | 0 | 317 | 211 | 0.367 |
|  |  |  | 1 | 116 | 73 |  |
|  |  |  | 2 | 9 | 14 |  |
| 1 | KIAA0090 | rs709683 | 0 | 164 | 119 | 0.928 |
|  |  |  | 1 | 221 | 136 |  |
|  |  |  | 2 | 57 | 45 |  |
| 1 | KIAA0090 | rs2294944 | 0 | 336 | 224 | 0.388 |
|  |  |  | 1 | 101 | 66 |  |
|  |  |  | 2 | 6 | 10 |  |
| 1 | KIAA0090 | rs10917277 | 0 | 243 | 152 | 0.080 |
|  |  |  | 1 | 172 | 115 |  |
|  |  |  | 2 | 28 | 32 |  |
| 1 | RIMS3 | rs12059305 | 0 | 289 | 214 | 0.067 |
|  |  |  | 1 | 137 | 78 |  |
|  |  |  | 2 | 14 | 6 |  |
| 1 | RIMS3 | rs17412556 | 0 | 323 | 229 | 0.341 |
|  |  |  | 1 | 112 | 64 |  |
|  |  |  | 2 | 8 | 6 |  |
| 1 | RIMS3 | rs7520333 | 0 | 128 | 101 | 0.866 |
|  |  |  | 1 | 229 | 131 |  |
|  |  |  | 2 | 82 | 67 |  |
| 1 | RIMS3 | rs570671 | 0 | 275 | 176 | 0.102 |
|  |  |  | 1 | 149 | 101 |  |
|  |  |  | 2 | 15 | 21 |  |
| 1 | RIMS3 | rs7553191 | 0 | 190 | 147 | 0.147 |
|  |  |  | 1 | 198 | 119 |  |
|  |  |  | 2 | 54 | 33 |  |
| 1 | RIMS3 | rs12562229 | 0 | 251 | 174 | 0.997 |
|  |  |  | 1 | 161 | 104 |  |
|  |  |  | 2 | 28 | 22 |  |
| 1 | RIMS3 | rs11208590 | 0 | 207 | 135 | 0.490 |
|  |  |  | 1 | 185 | 126 |  |
|  |  |  | 2 | 50 | 39 |  |
| 1 | RIMS3 | rs12728027 | 0 | 218 | 145 | 0.537 |
|  |  |  | 1 | 180 | 117 |  |
|  |  |  | 2 | 43 | 36 |  |
| 1 | ABCA4 | rs570926 | 0 | 162 | 116 | 0.832 |
|  |  |  | 1 | 217 | 136 |  |
|  |  |  | 2 | 63 | 46 |  |
| 1 | ABCA4 | rs1191236 | 0 | 243 | 169 | 0.388 |
|  |  |  | 1 | 167 | 118 |  |
|  |  |  | 2 | 31 | 13 |  |
| 1 | ABCA4 | rs3789434 | 0 | 361 | 243 | 0.793 |
|  |  |  | 1 | 78 | 52 |  |
|  |  |  | 2 | 5 | 5 |  |
| 1 | ABCA4 | rs2297634 | 0 | 116 | 81 | 0.713 |
|  |  |  | 1 | 215 | 149 |  |
|  |  |  | 2 | 107 | 69 |  |
| 1 | ABCA4 | rs3789444 | 0 | 273 | 161 | 0.052 |
|  |  |  | 1 | 149 | 122 |  |
|  |  |  | 2 | 18 | 14 |  |
| 1 | ABCA4 | rs7535005 | 0 | 236 | 159 | 0.540 |
|  |  |  | 1 | 166 | 127 |  |
|  |  |  | 2 | 35 | 13 |  |
| 1 | ABCA4 | rs3761906 | 0 | 375 | 259 | 0.487 |
|  |  |  | 1 | 67 | 38 |  |
|  |  |  | 2 | 2 | 2 |  |
| 1 | KLHL20 / CENPL / DARS2 | rs7550036 | 0 | 443 | 296 | 0.110 |
|  |  |  | 1 | 1 | 4 |  |
|  |  |  | 2 | 0 | 0 |  |
| 1 | KLHL20 / CENPL / DARS2 | rs6696680 | 0 | 209 | 147 | 0.160 |
|  |  |  | 1 | 192 | 138 |  |
|  |  |  | 2 | 43 | 15 |  |
| 1 | KLHL20 / CENPL / DARS2 | rs7536773 | 0 | 442 | 296 | 0.208 |
|  |  |  | 1 | 2 | 4 |  |
|  |  |  | 2 | 0 | 0 |  |
| 1 | KLHL20 / CENPL / DARS2 | rs6692452 | 0 | 441 | 296 | 0.371 |
|  |  |  | 1 | 3 | 4 |  |
|  |  |  | 2 | 0 | 0 |  |
| 1 | KLHL20 / CENPL / DARS2 | rs7552741 | 0 | 210 | 147 | 0.123 |
|  |  |  | 1 | 188 | 134 |  |
|  |  |  | 2 | 45 | 15 |  |
| 1 | KLHL20 / CENPL / DARS2 | rs2068871 | 0 | 362 | 246 | 0.856 |
|  |  |  | 1 | 75 | 52 |  |
|  |  |  | 2 | 5 | 2 |  |
| 1 | KLHL20 / CENPL / DARS2 | rs6691327 | 0 | 443 | 296 | 0.110 |
|  |  |  | 1 | 1 | 4 |  |
|  |  |  | 2 | 0 | 0 |  |
| 1 | KLHL20 / CENPL / DARS2 | rs9425756 | 0 | 245 | 166 | 0.324 |
|  |  |  | 1 | 165 | 124 |  |
|  |  |  | 2 | 34 | 10 |  |
| 1 | KIF21B | rs296569 | 0 | 203 | 160 | 0.678 |
|  |  |  | 1 | 99 | 84 |  |
|  |  |  | 2 | 17 | 8 |  |
| 1 | KIF21B | rs2275485 | 0 | 282 | 199 | 0.237 |
|  |  |  | 1 | 142 | 96 |  |
|  |  |  | 2 | 16 | 4 |  |
| 1 | KIF21B | rs2297909 | 0 | 216 | 140 | 0.339 |
|  |  |  | 1 | 196 | 132 |  |
|  |  |  | 2 | 28 | 26 |  |
| 1 | KIF21B | rs7536000 | 0 | 84 | 63 | 0.995 |
|  |  |  | 1 | 162 | 135 |  |
|  |  |  | 2 | 74 | 55 |  |
| 1 | KIF21B | rs10920091^a^ | 0 | 200 | 124 | 0.512 |
|  |  |  | 1 | 215 | 144 |  |
|  |  |  | 2 | 46 | 33 |  |
| 1 | KIF21B | rs12121970 | 0 | 334 | 230 | 0.364 |
|  |  |  | 1 | 99 | 70 |  |
|  |  |  | 2 | 9 | 0 |  |
| 1 | KIF21B | rs957957 | 0 | 129 | 73 | 0.466 |
|  |  |  | 1 | 217 | 81 |  |
|  |  |  | 2 | 92 | 12 |  |
| 1 | C1orf116 / YOD1 / PFKFB2 | rs4301633 | 0 | 322 | 207 | 0.148 |
|  |  |  | 1 | 112 | 81 |  |
|  |  |  | 2 | 9 | 12 |  |
| 1 | C1orf116 / YOD1 / PFKFB2 | rs12403925 | 0 | 332 | 216 | 0.479 |
|  |  |  | 1 | 104 | 77 |  |
|  |  |  | 2 | 7 | 5 |  |
| 1 | C1orf116 / YOD1 / PFKFB2 | rs17258711 | 0 | 135 | 96 | 0.916 |
|  |  |  | 1 | 228 | 145 |  |
|  |  |  | 2 | 81 | 58 |  |
| 1 | C1orf116 / YOD1 / PFKFB2 | rs12123107 | 0 | 133 | 95 | 0.874 |
|  |  |  | 1 | 225 | 145 |  |
|  |  |  | 2 | 82 | 58 |  |
| 1 | C1orf116 / YOD1 / PFKFB2 | rs2054780 | 0 | 330 | 217 | 0.940 |
|  |  |  | 1 | 103 | 79 |  |
|  |  |  | 2 | 11 | 3 |  |
| 1 | C1orf116 / YOD1 / PFKFB2 | rs2306714 | 0 | 135 | 96 | 0.948 |
|  |  |  | 1 | 223 | 142 |  |
|  |  |  | 2 | 82 | 61 |  |
| 1 | C1orf116 / YOD1 / PFKFB2 | rs873140 | 0 | 135 | 98 | 0.854 |
|  |  |  | 1 | 222 | 142 |  |
|  |  |  | 2 | 82 | 59 |  |
| 1 | C1orf116 / YOD1 / PFKFB2 | rs11120148 | 0 | 130 | 95 | 0.886 |
|  |  |  | 1 | 228 | 148 |  |
|  |  |  | 2 | 78 | 57 |  |
| 1 | C1orf116 / YOD1 / PFKFB2 | rs17258746 | 0 | 332 | 220 | 0.587 |
|  |  |  | 1 | 103 | 76 |  |
|  |  |  | 2 | 6 | 4 |  |
| 1 | C1orf116 / YOD1 / PFKFB2 | rs3748671 | 0 | 143 | 89 | 0.646 |
|  |  |  | 1 | 205 | 146 |  |
|  |  |  | 2 | 95 | 64 |  |
| 1 | C1orf116 / YOD1 / PFKFB2 | rs4142866 | 0 | 101 | 72 | 0.522 |
|  |  |  | 1 | 240 | 165 |  |
|  |  |  | 2 | 102 | 63 |  |
| 1 | C4BPB / C4BPA | rs2353545 | 0 | 104 | 66 | 0.524 |
|  |  |  | 1 | 239 | 161 |  |
|  |  |  | 2 | 100 | 73 |  |
| 1 | C4BPB / C4BPA | rs7536934 | 0 | 440 | 298 | 0.727 |
|  |  |  | 1 | 4 | 2 |  |
|  |  |  | 2 | 0 | 0 |  |
| 1 | C4BPB / C4BPA | rs8942 | 0 | 292 | 196 | 0.934 |
|  |  |  | 1 | 135 | 95 |  |
|  |  |  | 2 | 15 | 9 |  |
| 1 | C4BPB / C4BPA | rs17020634 | 0 | 373 | 265 | 0.135 |
|  |  |  | 1 | 69 | 34 |  |
|  |  |  | 2 | 1 | 1 |  |
| 1 | C4BPB / C4BPA | rs11120211 | 0 | 372 | 265 | 0.116 |
|  |  |  | 1 | 70 | 34 |  |
|  |  |  | 2 | 1 | 1 |  |
| 1 | C4BPB / C4BPA | rs17020993 | 0 | FAILED SNP | FAILED SNP | FAILED SNP |
|  |  |  | 1 |  |  |  |
|  |  |  | 2 |  |  |  |
| 1 | C4BPB / C4BPA | rs6682764 | 0 | 184 | 134 | 0.235 |
|  |  |  | 1 | 194 | 131 |  |
|  |  |  | 2 | 65 | 35 |  |
| 1 | C4BPB / C4BPA | rs4844573 | 0 | 213 | 135 | 0.281 |
|  |  |  | 1 | 179 | 123 |  |
|  |  |  | 2 | 51 | 42 |  |
| 1 | C4BPB / C4BPA | rs4571969 | 0 | 265 | 174 | 0.686 |
|  |  |  | 1 | 153 | 108 |  |
|  |  |  | 2 | 26 | 18 |  |
| 1 | LOC127602 | rs12038394 | 0 | 159 | 85 | 0.024 |
|  |  |  | 1 | 214 | 157 |  |
|  |  |  | 2 | 68 | 58 |  |
| 1 | LOC127602 | rs3128655 | 0 | 355 | 229 | 0.342 |
|  |  |  | 1 | 78 | 67 |  |
|  |  |  | 2 | 9 | 4 |  |
| 1 | LOC127602 | rs4275419 | 0 | 441 | 298 | 0.988 |
|  |  |  | 1 | 3 | 2 |  |
|  |  |  | 2 | 0 | 0 |  |
| 1 | LOC127602 | rs1871570 | 0 | 113 | 99 | 0.003 |
|  |  |  | 1 | 225 | 155 |  |
|  |  |  | 2 | 104 | 46 |  |
| 1 | LOC127602 | rs4308947 | 0 | 206 | 121 | 0.225 |
|  |  |  | 1 | 189 | 147 |  |
|  |  |  | 2 | 48 | 32 |  |
| 1 | LOC127602 | rs581675 | 0 | 366 | 238 | 0.283 |
|  |  |  | 1 | 73 | 59 |  |
|  |  |  | 2 | 4 | 3 |  |
| 1 | LOC127602 | rs12041072 | 0 | 309 | 200 | 0.367 |
|  |  |  | 1 | 125 | 91 |  |
|  |  |  | 2 | 9 | 8 |  |
| 1 | LOC127602 | rs3856145 | 0 | 157 | 92 | 0.311 |
|  |  |  | 1 | 206 | 151 |  |
|  |  |  | 2 | 80 | 56 |  |
| 1 | LOC127602 | rs12184324 | 0 | 409 | 275 | 0.707 |
|  |  |  | 1 | 35 | 24 |  |
|  |  |  | 2 | 0 | 1 |  |
| 1 | LOC127602 | rs6667999 | 0 | 153 | 93 | 0.478 |
|  |  |  | 1 | 210 | 150 |  |
|  |  |  | 2 | 80 | 55 |  |
| 1 | LOC127602 | rs7527925 | 0 | 154 | 92 | 0.380 |
|  |  |  | 1 | 209 | 150 |  |
|  |  |  | 2 | 80 | 56 |  |
| 1 | C1orf145 / OBSCN / KIAA1639 | rs1771487 | 0 | 126 | 99 | 0.176 |
|  |  |  | 1 | 160 | 108 |  |
|  |  |  | 2 | 34 | 46 |  |
| 1 | C1orf145 / OBSCN / KIAA1639 | rs2776857 | 0 | 414 | 274 | 0.185 |
|  |  |  | 1 | 28 | 25 |  |
|  |  |  | 2 | 0 | 1 |  |
| 1 | C1orf145 / OBSCN / KIAA1639 | rs4653544 | 0 | 247 | 164 | 0.978 |
|  |  |  | 1 | 167 | 120 |  |
|  |  |  | 2 | 29 | 16 |  |
| 1 | C1orf145 / OBSCN / KIAA1639 | rs424645 | 0 | FAILED SNP | FAILED SNP | FAILED SNP |
|  |  |  | 1 |  |  |  |
|  |  |  | 2 |  |  |  |
| 1 | C1orf145 / OBSCN / KIAA1639 | rs435776 | 0 | 86 | 60 | 0.237 |
|  |  |  | 1 | 164 | 128 |  |
|  |  |  | 2 | 70 | 65 |  |
| 1 | C1orf145 / OBSCN / KIAA1639 | rs54108 | 0 | 87 | 60 | 0.200 |
|  |  |  | 1 | 165 | 129 |  |
|  |  |  | 2 | 68 | 64 |  |
| 1 | C1orf145 / OBSCN / KIAA1639 | rs4653942 | 0 | 213 | 170 | 0.368 |
|  |  |  | 1 | 100 | 65 |  |
|  |  |  | 2 | 7 | 18 |  |
| 1 | C1orf145 / OBSCN / KIAA1639 | rs1188729 | 0 | 216 | 141 | 0.693 |
|  |  |  | 1 | 177 | 129 |  |
|  |  |  | 2 | 41 | 27 |  |
| 1 | C1orf145 / OBSCN / KIAA1639 | rs373610 | 0 | 157 | 109 | 0.477 |
|  |  |  | 1 | 129 | 107 |  |
|  |  |  | 2 | 28 | 21 |  |
| 1 | C1orf145 / OBSCN / KIAA1639 | rs369909 | 0 | 125 | 94 | 0.819 |
|  |  |  | 1 | 232 | 142 |  |
|  |  |  | 2 | 80 | 60 |  |
| 1 | C1orf145 / OBSCN / KIAA1639 | rs1188710 | 0 | 95 | 67 | 0.170 |
|  |  |  | 1 | 167 | 128 |  |
|  |  |  | 2 | 58 | 58 |  |
| 1 | C1orf145 / OBSCN / KIAA1639 | rs3795814 | 0 | 263 | 219 | 0.201 |
|  |  |  | 1 | 55 | 32 |  |
|  |  |  | 2 | 2 | 2 |  |
| 1 | C1orf145 / OBSCN / KIAA1639 | rs12085738 | 0 | 266 | 218 | 0.378 |
|  |  |  | 1 | 52 | 33 |  |
|  |  |  | 2 | 2 | 2 |  |
| 2 | ANTXR1 | rs12465132 | 0 | 402 | 277 | 0.431 |
|  |  |  | 1 | 38 | 19 |  |
|  |  |  | 2 | 2 | 2 |  |
| 2 | ANTXR1 | rs1106518 | 0 | 249 | 162 | 0.789 |
|  |  |  | 1 | 158 | 117 |  |
|  |  |  | 2 | 34 | 20 |  |
| 2 | ANTXR1 | rs4405800 | 0 | 350 | 223 | 0.156 |
|  |  |  | 1 | 88 | 71 |  |
|  |  |  | 2 | 4 | 4 |  |
| 2 | ANTXR1 | rs6710260 | 0 | 312 | 216 | 0.695 |
|  |  |  | 1 | 121 | 78 |  |
|  |  |  | 2 | 6 | 4 |  |
| 2 | ANTXR1 | rs13424360 | 0 | 328 | 228 | 0.713 |
|  |  |  | 1 | 108 | 67 |  |
|  |  |  | 2 | 4 | 4 |  |
| 2 | ANTXR1 | rs12611797 | 0 | 201 | 136 | 0.866 |
|  |  |  | 1 | 194 | 130 |  |
|  |  |  | 2 | 47 | 34 |  |
| 2 | ANTXR1 | rs12713671 | 0 | 156 | 99 | 0.711 |
|  |  |  | 1 | 207 | 158 |  |
|  |  |  | 2 | 77 | 40 |  |
| 2 | intragenic | rs2358259 | 0 | 442 | 298 | 0.694 |
|  |  |  | 1 | 2 | 2 |  |
|  |  |  | 2 | 0 | 0 |  |
| 2 | LOC643997 | rs16861806 | 0 | 388 | 259 | 0.426 |
|  |  |  | 1 | 55 | 37 |  |
|  |  |  | 2 | 1 | 4 |  |
| 2 | LOC643997 | rs4972578 | 0 | 147 | 111 | 0.268 |
|  |  |  | 1 | 220 | 144 |  |
|  |  |  | 2 | 75 | 45 |  |
| 3 | intragenic | rs2055451 | 0 | 231 | 146 | 0.106 |
|  |  |  | 1 | 178 | 115 |  |
|  |  |  | 2 | 35 | 38 |  |
| 3 | intragenic | rs1426054 | 0 | 112 | 73 | 0.849 |
|  |  |  | 1 | 217 | 157 |  |
|  |  |  | 2 | 112 | 70 |  |
| 3 | intragenic | rs11926120 | 0 | 442 | 299 | 0.805 |
|  |  |  | 1 | 2 | 1 |  |
|  |  |  | 2 | 0 | 0 |  |
| 4 | GRSF1 | rs13112870 | 0 | 394 | 250 | 0.034 |
|  |  |  | 1 | 46 | 47 |  |
|  |  |  | 2 | 3 | 3 |  |
| 4 | GRSF1 | rs3775729 | 0 | 408 | 273 | 0.409 |
|  |  |  | 1 | 34 | 24 |  |
|  |  |  | 2 | 1 | 3 |  |
| 4 | GRSF1 | rs4694348 | 0 | 406 | 272 | 0.419 |
|  |  |  | 1 | 35 | 23 |  |
|  |  |  | 2 | 0 | 3 |  |
| 4 | MGC46496 | rs1911792 | 0 | 135 | 101 | 0.883 |
|  |  |  | 1 | 216 | 131 |  |
|  |  |  | 2 | 90 | 68 |  |
| 4 | MGC46496 | rs1911793 | 0 | 281 | 201 | 0.477 |
|  |  |  | 1 | 143 | 85 |  |
|  |  |  | 2 | 17 | 13 |  |
| 4 | MGC46496 | rs1508459 | 0 | 437 | 297 | 0.695 |
|  |  |  | 1 | 4 | 3 |  |
|  |  |  | 2 | 1 | 0 |  |
| 4 | MGC46496 | rs10213173 | 0 | 214 | 138 | 0.347 |
|  |  |  | 1 | 181 | 125 |  |
|  |  |  | 2 | 45 | 37 |  |
| 4 | MGC46496 | rs6837669 | 0 | 149 | 87 | 0.360 |
|  |  |  | 1 | 198 | 147 |  |
|  |  |  | 2 | 96 | 66 |  |
| 4 | MGC46496 | rs936204 | 0 | 335 | 226 | 0.996 |
|  |  |  | 1 | 101 | 69 |  |
|  |  |  | 2 | 8 | 5 |  |
| 4 | MGC46496 | rs956424 | 0 | 341 | 239 | 0.419 |
|  |  |  | 1 | 99 | 56 |  |
|  |  |  | 2 | 4 | 4 |  |
| 4 | MGC46496 | rs783959 | 0 | 118 | 89 | 0.262 |
|  |  |  | 1 | 216 | 146 |  |
|  |  |  | 2 | 106 | 63 |  |
| 4 | MGC46496 | rs880471 | 0 | 208 | 142 | 0.612 |
|  |  |  | 1 | 196 | 122 |  |
|  |  |  | 2 | 40 | 36 |  |
| 4 | MGC46496 | rs1426671 | 0 | 386 | 254 | 0.294 |
|  |  |  | 1 | 55 | 43 |  |
|  |  |  | 2 | 1 | 2 |  |
| 4 | MGC46496 | rs6817529 | 0 | 166 | 108 | 0.223 |
|  |  |  | 1 | 217 | 136 |  |
|  |  |  | 2 | 61 | 56 |  |
| 4 | MGC46496 | rs2903150 | 0 | 164 | 107 | 0.200 |
|  |  |  | 1 | 215 | 135 |  |
|  |  |  | 2 | 61 | 57 |  |
| 4 | MGC46496 | rs17558193 | 0 | 166 | 108 | 0.189 |
|  |  |  | 1 | 214 | 134 |  |
|  |  |  | 2 | 62 | 58 |  |
| 4 | MGC45800 | rs1447338 | 0 | 253 | 186 | 0.171 |
|  |  |  | 1 | 169 | 102 |  |
|  |  |  | 2 | 22 | 12 |  |
| 4 | MGC45800 | rs13129209 | 0 | 200 | 145 | 0.652 |
|  |  |  | 1 | 200 | 123 |  |
|  |  |  | 2 | 43 | 32 |  |
| 6 | GLO1 / DNAH8 | rs7604 | 0 | 346 | 237 | 0.786 |
|  |  |  | 1 | 91 | 59 |  |
|  |  |  | 2 | 6 | 4 |  |
| 6 | GLO1 / DNAH8 | rs3799703 | 0 | 132 | 81 | 0.549 |
|  |  |  | 1 | 216 | 154 |  |
|  |  |  | 2 | 93 | 64 |  |
| 6 | GLO1 / DNAH8 | rs2736654 | 0 | 130 | 87 | 0.611 |
|  |  |  | 1 | 229 | 150 |  |
|  |  |  | 2 | 83 | 63 |  |
| 6 | GLO1 / DNAH8 | rs3778443 | 0 | 394 | 263 | 0.484 |
|  |  |  | 1 | 47 | 34 |  |
|  |  |  | 2 | 2 | 3 |  |
| 6 | GLO1 / DNAH8 | rs10484854 | 0 | 246 | 159 | 0.462 |
|  |  |  | 1 | 169 | 119 |  |
|  |  |  | 2 | 27 | 21 |  |
| 6 | GLO1 / DNAH8 | rs17622621 | 0 | 172 | 115 | 0.822 |
|  |  |  | 1 | 213 | 143 |  |
|  |  |  | 2 | 56 | 40 |  |
| 6 | GLO1 / DNAH8 | rs1781735 | 0 | 119 | 70 | 0.246 |
|  |  |  | 1 | 228 | 158 |  |
|  |  |  | 2 | 94 | 71 |  |
| 6 | GLO1 / DNAH8 | rs1937780 | 0 | 163 | 112 | 0.944 |
|  |  |  | 1 | 218 | 145 |  |
|  |  |  | 2 | 60 | 43 |  |
| 6 | GLO1 / DNAH8 | rs1781731 | 0 | 363 | 242 | 0.946 |
|  |  |  | 1 | 68 | 52 |  |
|  |  |  | 2 | 8 | 2 |  |
| 6 | GLO1 / DNAH8 | rs12196682 | 0 | 326 | 212 | 0.468 |
|  |  |  | 1 | 105 | 79 |  |
|  |  |  | 2 | 13 | 9 |  |
| 6 | GLO1 / DNAH8 | rs1937781 | 0 | 179 | 133 | 0.073 |
|  |  |  | 1 | 203 | 140 |  |
|  |  |  | 2 | 62 | 27 |  |
| 6 | GLO1 / DNAH8 | rs9296260 | 0 | 270 | 170 | 0.460 |
|  |  |  | 1 | 150 | 116 |  |
|  |  |  | 2 | 23 | 13 |  |
| 6 | GLO1 / DNAH8 | rs7761815 | 0 | 272 | 170 | 0.393 |
|  |  |  | 1 | 149 | 117 |  |
|  |  |  | 2 | 23 | 13 |  |
| 6 | GLO1 / DNAH8 | rs1698998 | 0 | 324 | 200 | 0.070 |
|  |  |  | 1 | 114 | 93 |  |
|  |  |  | 2 | 6 | 6 |  |
| 6 | GLO1 / DNAH8 | rs6458067 | 0 | 127 | 95 | 0.164 |
|  |  |  | 1 | 210 | 148 |  |
|  |  |  | 2 | 104 | 57 |  |
| 6 | GLO1 / DNAH8 | rs1623375 | 0 | 135 | 73 | 0.056 |
|  |  |  | 1 | 219 | 155 |  |
|  |  |  | 2 | 89 | 72 |  |
| 6 | GLO1 / DNAH8 | rs17552381 | 0 | 228 | 163 | 0.161 |
|  |  |  | 1 | 172 | 118 |  |
|  |  |  | 2 | 43 | 18 |  |
| 6 | GLO1 / DNAH8 | rs1678674 | 0 | 419 | 277 | 0.284 |
|  |  |  | 1 | 24 | 22 |  |
|  |  |  | 2 | 0 | 0 |  |
| 6 | GLO1 / DNAH8 | rs9357283 | 0 | 332 | 238 | 0.307 |
|  |  |  | 1 | 104 | 55 |  |
|  |  |  | 2 | 7 | 7 |  |
| 6 | GLO1 / DNAH8 | rs10484847 | 0 | 292 | 222 | 0.039 |
|  |  |  | 1 | 128 | 64 |  |
|  |  |  | 2 | 22 | 13 |  |
| 6 | AHI1 | rs1052502 | 0 | 377 | 250 | 0.688 |
|  |  |  | 1 | 63 | 48 |  |
|  |  |  | 2 | 3 | 1 |  |
| 6 | AHI1 | rs9285480 | 0 | 405 | 272 | 0.661 |
|  |  |  | 1 | 35 | 28 |  |
|  |  |  | 2 | 1 | 0 |  |
| 6 | AHI1 | rs11154798 | 0 | 126 | 114 | 0.045 |
|  |  |  | 1 | 229 | 129 |  |
|  |  |  | 2 | 89 | 57 |  |
| 6 | AHI1 | rs7759971 | 0 | 157 | 131 | 0.082 |
|  |  |  | 1 | 216 | 123 |  |
|  |  |  | 2 | 70 | 45 |  |
| 6 | AHI1 | rs2614273 | 0 | 375 | 243 | 0.328 |
|  |  |  | 1 | 61 | 54 |  |
|  |  |  | 2 | 7 | 3 |  |
| 6 | AHI1 | rs9373131 | 0 | FAILED SNP | FAILED SNP | FAILED SNP |
|  |  |  | 1 |  |  |  |
|  |  |  | 2 |  |  |  |
| 6 | TXLNB | rs9484252 | 0 | 438 | 293 | 0.322 |
|  |  |  | 1 | 6 | 7 |  |
|  |  |  | 2 | 0 | 0 |  |
| 6 | TXLNB | rs10499208 | 0 | 276 | 188 | 0.939 |
|  |  |  | 1 | 142 | 95 |  |
|  |  |  | 2 | 25 | 17 |  |
| 6 | TXLNB | rs9321711 | 0 | 372 | 252 | 0.865 |
|  |  |  | 1 | 69 | 48 |  |
|  |  |  | 2 | 2 | 0 |  |
| 6 | TXLNB | rs9495391 | 0 | 176 | 126 | 0.896 |
|  |  |  | 1 | 219 | 128 |  |
|  |  |  | 2 | 49 | 43 |  |
| 6 | TXLNB | rs17068468 | 0 | 332 | 207 | 0.175 |
|  |  |  | 1 | 98 | 85 |  |
|  |  |  | 2 | 14 | 8 |  |
| 6 | TXLNB | rs7762039 | 0 | 280 | 179 | 0.396 |
|  |  |  | 1 | 139 | 104 |  |
|  |  |  | 2 | 24 | 17 |  |
| 6 | TXLNB | rs6930482 | 0 | 390 | 267 | 0.931 |
|  |  |  | 1 | 49 | 30 |  |
|  |  |  | 2 | 0 | 2 |  |
| 6 | TXLNB | rs6927460 | 0 | 254 | 164 | 0.657 |
|  |  |  | 1 | 154 | 114 |  |
|  |  |  | 2 | 35 | 22 |  |
| 7 | CARD4 | rs5743373 | 0 | 412 | 278 | 0.948 |
|  |  |  | 1 | 32 | 22 |  |
|  |  |  | 2 | 0 | 0 |  |
| 7 | CARD4 | rs5743371 | 0 | 412 | 276 | 0.803 |
|  |  |  | 1 | 32 | 23 |  |
|  |  |  | 2 | 0 | 0 |  |
| 7 | NOD1 | rs5743369 | 0 | 338 | 220 | 0.879 |
|  |  |  | 1 | 91 | 76 |  |
|  |  |  | 2 | 14 | 3 |  |
| 7 | NOD1 | rs2907749 | 0 | 210 | 148 | 0.610 |
|  |  |  | 1 | 186 | 122 |  |
|  |  |  | 2 | 47 | 30 |  |
| 7 | NOD1 | rs2075820 | 0 | 264 | 166 | 0.311 |
|  |  |  | 1 | 145 | 113 |  |
|  |  |  | 2 | 28 | 19 |  |
| 7 | NOD1 | rs2906766 | 0 | 240 | 126 | 0.009 |
|  |  |  | 1 | 158 | 140 |  |
|  |  |  | 2 | 43 | 32 |  |
| 7 | NOD1 | rs2906773 | 0 | 164 | 121 | 0.274 |
|  |  |  | 1 | 210 | 138 |  |
|  |  |  | 2 | 69 | 40 |  |
| 7 | NOD1 | rs730360 | 0 | 282 | 213 | 0.080 |
|  |  |  | 1 | 139 | 73 |  |
|  |  |  | 2 | 20 | 13 |  |
| 7 | NOD1 | rs4722988 | 0 | 300 | 224 | 0.072 |
|  |  |  | 1 | 129 | 68 |  |
|  |  |  | 2 | 13 | 8 |  |
| 7 | NOD1 | rs6963954 | 0 | 396 | 252 | 0.016 |
|  |  |  | 1 | 42 | 45 |  |
|  |  |  | 2 | 2 | 3 |  |
| 7 | NOD1 | rs2256023 | 0 | 144 | 98 | 0.762 |
|  |  |  | 1 | 228 | 148 |  |
|  |  |  | 2 | 72 | 54 |  |
| 7 | ADCYAP1R1 | rs10241138 | 0 | 325 | 237 | 0.105 |
|  |  |  | 1 | 108 | 58 |  |
|  |  |  | 2 | 9 | 5 |  |
| 7 | ADCYAP1R1 | rs3779247 | 0 | FAILED SNP | FAILED SNP | FAILED SNP |
|  |  |  | 1 |  |  |  |
|  |  |  | 2 |  |  |  |
| 7 | ADCYAP1R1 | rs1006622 | 0 | 126 | 82 | 0.329 |
|  |  |  | 1 | 224 | 141 |  |
|  |  |  | 2 | 94 | 76 |  |
| 7 | ADCYAP1R1 | rs887703 | 0 | 187 | 115 | 0.296 |
|  |  |  | 1 | 197 | 138 |  |
|  |  |  | 2 | 59 | 45 |  |
| 7 | ADCYAP1R1 | rs2267730 | 0 | 132 | 76 | 0.146 |
|  |  |  | 1 | 207 | 145 |  |
|  |  |  | 2 | 99 | 77 |  |
| 7 | ADCYAP1R1 | rs2267742 | 0 | 371 | 258 | 0.387 |
|  |  |  | 1 | 66 | 37 |  |
|  |  |  | 2 | 1 | 1 |  |
| 7 | ADCYAP1R1 | rs741057 | 0 | 158 | 132 | 0.546 |
|  |  |  | 1 | 138 | 103 |  |
|  |  |  | 2 | 24 | 18 |  |
| 7 | NAPE-PLD | rs1968199 | 0 | 442 | 299 | 0.619 |
|  |  |  | 1 | 1 | 1 |  |
|  |  |  | 2 | 1 | 0 |  |
| 7 | PLXNA4B | rs10268061 | 0 | 384 | 250 | 0.169 |
|  |  |  | 1 | 56 | 46 |  |
|  |  |  | 2 | 3 | 4 |  |
| 7 | PLXNA4B | rs2341823 | 0 | 365 | 262 | 0.042 |
|  |  |  | 1 | 73 | 35 |  |
|  |  |  | 2 | 4 | 1 |  |
| 7 | PLXNA4B | rs156949 | 0 | 329 | 236 | 0.138 |
|  |  |  | 1 | 106 | 61 |  |
|  |  |  | 2 | 8 | 3 |  |
| 7 | PLXNA4B | rs10808265 | 0 | 146 | 94 | 0.734 |
|  |  |  | 1 | 216 | 150 |  |
|  |  |  | 2 | 79 | 54 |  |
| 7 | PLXNA4B | rs156963 | 0 | 168 | 113 | 0.939 |
|  |  |  | 1 | 208 | 144 |  |
|  |  |  | 2 | 65 | 42 |  |
| 7 | PLXNA4B | rs885736 | 0 | 114 | 80 | 0.747 |
|  |  |  | 1 | 227 | 148 |  |
|  |  |  | 2 | 102 | 67 |  |
| 7 | PLXNA4B | rs12707045 | 0 | 292 | 199 | 0.765 |
|  |  |  | 1 | 134 | 93 |  |
|  |  |  | 2 | 16 | 8 |  |
| 7 | PLXNA4B | rs9649579 | 0 | 147 | 107 | 0.415 |
|  |  |  | 1 | 221 | 148 |  |
|  |  |  | 2 | 71 | 43 |  |
| 7 | PLXNA4B | rs12707048 | 0 | 283 | 202 | 0.203 |
|  |  |  | 1 | 147 | 93 |  |
|  |  |  | 2 | 14 | 5 |  |
| 7 | PLXNA4B | rs12707049 | 0 | 111 | 80 | 0.610 |
|  |  |  | 1 | 226 | 149 |  |
|  |  |  | 2 | 105 | 68 |  |
| 7 | PLXNA4B | rs1499300 | 0 | 296 | 226 | 0.007 |
|  |  |  | 1 | 135 | 72 |  |
|  |  |  | 2 | 11 | 2 |  |
| 7 | PLXNA4B | rs10273006 | 0 | 143 | 90 | 0.653 |
|  |  |  | 1 | 219 | 155 |  |
|  |  |  | 2 | 81 | 55 |  |
| 7 | PLXNA4B | rs6966705 | 0 | 152 | 94 | 0.372 |
|  |  |  | 1 | 215 | 150 |  |
|  |  |  | 2 | 74 | 55 |  |
| 7 | PLXNA4B | rs11773117 | 0 | 318 | 235 | 0.010 |
|  |  |  | 1 | 113 | 65 |  |
|  |  |  | 2 | 12 | 0 |  |
| 7 | PLXNA4B | rs11508530 | 0 | 111 | 85 | 0.077 |
|  |  |  | 1 | 222 | 158 |  |
|  |  |  | 2 | 109 | 56 |  |
| 7 | PLXNA4B | rs6965827 | 0 | 205 | 145 | 0.773 |
|  |  |  | 1 | 192 | 122 |  |
|  |  |  | 2 | 46 | 33 |  |
| 9 | SLC28A3 | rs7042987 | 0 | 364 | 257 | 0.119 |
|  |  |  | 1 | 75 | 39 |  |
|  |  |  | 2 | 3 | 1 |  |
| 9 | SLC28A3 | rs885004 | 0 | 321 | 239 | 0.030 |
|  |  |  | 1 | 118 | 53 |  |
|  |  |  | 2 | 5 | 5 |  |
| 9 | SLC28A3 | rs10868138 | 0 | 369 | 260 | 0.160 |
|  |  |  | 1 | 69 | 38 |  |
|  |  |  | 2 | 4 | 1 |  |
| 9 | SLC28A3 | rs4877837 | 0 | 309 | 212 | 0.942 |
|  |  |  | 1 | 125 | 80 |  |
|  |  |  | 2 | 9 | 8 |  |
| 9 | SLC28A3 | rs7867504 | 0 | 202 | 154 | 0.084 |
|  |  |  | 1 | 188 | 120 |  |
|  |  |  | 2 | 50 | 25 |  |
| 9 | SLC28A3 | rs9792674 | 0 | 228 | 157 | 0.732 |
|  |  |  | 1 | 167 | 114 |  |
|  |  |  | 2 | 47 | 29 |  |
| 9 | SLC28A3 | rs10780661 | 0 | 292 | 210 | 0.112 |
|  |  |  | 1 | 124 | 81 |  |
|  |  |  | 2 | 26 | 9 |  |
| 9 | SLC28A3 | rs4877839 | 0 | 371 | 240 | 0.164 |
|  |  |  | 1 | 68 | 57 |  |
|  |  |  | 2 | 3 | 3 |  |
| 9 | SLC28A3 | rs4877844 | 0 | 296 | 210 | 0.333 |
|  |  |  | 1 | 132 | 81 |  |
|  |  |  | 2 | 16 | 9 |  |
| 9 | SLC28A3 | rs7035188 | 0 | 372 | 266 | 0.058 |
|  |  |  | 1 | 64 | 32 |  |
|  |  |  | 2 | 7 | 2 |  |
| 9 | SLC28A3 | rs11140522 | 0 | 359 | 241 | 0.811 |
|  |  |  | 1 | 77 | 54 |  |
|  |  |  | 2 | 7 | 5 |  |
| 9 | SLC28A3 | rs4877845 | 0 | 258 | 188 | 0.148 |
|  |  |  | 1 | 154 | 96 |  |
|  |  |  | 2 | 31 | 15 |  |
| 9 | SLC28A3 | rs4305983 | 0 | 233 | 162 | 0.413 |
|  |  |  | 1 | 170 | 117 |  |
|  |  |  | 2 | 40 | 20 |  |
| 9 | SLC28A3 | rs4877847 | 0 | 152 | 98 | 0.546 |
|  |  |  | 1 | 190 | 148 |  |
|  |  |  | 2 | 100 | 53 |  |
| 9 | SLC28A3 | rs4877851 | 0 | 331 | 223 | 0.608 |
|  |  |  | 1 | 106 | 68 |  |
|  |  |  | 2 | 5 | 8 |  |
| 9 | SLC28A3 | rs7035753 | 0 | 182 | 129 | 0.088 |
|  |  |  | 1 | 188 | 141 |  |
|  |  |  | 2 | 73 | 29 |  |
| 9 | SLC28A3 | rs11140542 | 0 | 281 | 195 | 0.635 |
|  |  |  | 1 | 148 | 94 |  |
|  |  |  | 2 | 15 | 10 |  |
| 9 | SLC28A3 | rs17087152 | 0 | 421 | 289 | 0.335 |
|  |  |  | 1 | 23 | 11 |  |
|  |  |  | 2 | 0 | 0 |  |
| 9 | SLC28A3 | rs7030016 | 0 | 238 | 164 | 0.852 |
|  |  |  | 1 | 171 | 107 |  |
|  |  |  | 2 | 33 | 28 |  |
| 10 | FRAT1 / FRAT2 | rs915196 | 0 | FAILED SNP | FAILED SNP | FAILED SNP |
|  |  |  | 1 |  |  |  |
|  |  |  | 2 |  |  |  |
| 11 | FADS2 / FADS3 | rs1535 | 0 | 192 | 114 | 0.123 |
|  |  |  | 1 | 196 | 144 |  |
|  |  |  | 2 | 52 | 42 |  |
| 11 | FADS2 / FADS3 | rs2845573 | 0 | 384 | 241 | 0.019 |
|  |  |  | 1 | 57 | 54 |  |
|  |  |  | 2 | 3 | 5 |  |
| 11 | FADS2 / FADS3 | rs2727271 | 0 | 355 | 218 | 0.041 |
|  |  |  | 1 | 75 | 70 |  |
|  |  |  | 2 | 10 | 8 |  |
| 11 | FADS2 / FADS3 | rs174589 | 0 | 277 | 177 | 0.569 |
|  |  |  | 1 | 145 | 112 |  |
|  |  |  | 2 | 21 | 11 |  |
| 11 | FADS2 / FADS3 | rs174602 | 0 | 287 | 172 | 0.034 |
|  |  |  | 1 | 141 | 113 |  |
|  |  |  | 2 | 15 | 15 |  |
| 11 | FADS2 / FADS3 | rs174605 | 0 | 235 | 140 | 0.352 |
|  |  |  | 1 | 162 | 131 |  |
|  |  |  | 2 | 45 | 26 |  |
| 11 | FADS2 / FADS3 | rs11230815 | 0 | 343 | 233 | 0.959 |
|  |  |  | 1 | 93 | 61 |  |
|  |  |  | 2 | 7 | 6 |  |
| 11 | FADS2 / FADS3 | rs1000778 | 0 | 255 | 160 | 0.411 |
|  |  |  | 1 | 155 | 119 |  |
|  |  |  | 2 | 30 | 19 |  |
| 11 | PHCA | rs11602065 | 0 | 123 | 90 | 0.932 |
|  |  |  | 1 | 238 | 145 |  |
|  |  |  | 2 | 81 | 63 |  |
| 11 | PHCA | rs11237005 | 0 | 391 | 262 | 0.693 |
|  |  |  | 1 | 49 | 37 |  |
|  |  |  | 2 | 2 | 1 |  |
| 11 | PHCA | rs4379869 | 0 | 220 | 158 | 0.415 |
|  |  |  | 1 | 197 | 124 |  |
|  |  |  | 2 | 27 | 17 |  |
| 11 | PHCA | rs681164 | 0 | 376 | 254 | 0.791 |
|  |  |  | 1 | 65 | 43 |  |
|  |  |  | 2 | 2 | 3 |  |
| 11 | PHCA | rs3740767 | 0 | 236 | 174 | 0.243 |
|  |  |  | 1 | 181 | 110 |  |
|  |  |  | 2 | 26 | 16 |  |
| 11 | PHCA | rs6592695 | 0 | FAILED SNP | FAILED SNP | FAILED SNP |
|  |  |  | 1 |  |  |  |
|  |  |  | 2 |  |  |  |
| 11 | PHCA | rs17135325 | 0 | 349 | 231 | 0.623 |
|  |  |  | 1 | 88 | 64 |  |
|  |  |  | 2 | 7 | 5 |  |
| 12 | LOC651030 | rs7316255 | 0 | 439 | 297 | 0.872 |
|  |  |  | 1 | 5 | 3 |  |
|  |  |  | 2 | 0 | 0 |  |
| 12 | LOC641695 | rs9669322 | 0 | 178 | 126 | 0.653 |
|  |  |  | 1 | 191 | 124 |  |
|  |  |  | 2 | 73 | 48 |  |
| 13 | intragenic | rs9514252 | 0 | 114 | 84 | 0.180 |
|  |  |  | 1 | 226 | 156 |  |
|  |  |  | 2 | 103 | 56 |  |
| 13 | intragenic | rs7996685 | 0 | 391 | 257 | 0.295 |
|  |  |  | 1 | 51 | 42 |  |
|  |  |  | 2 | 1 | 1 |  |
| 14 | BMP4 | rs8005200 | 0 | 440 | 297 | 0.890 |
|  |  |  | 1 | 4 | 3 |  |
|  |  |  | 2 | 0 | 0 |  |
| 14 | NAT12 | rs11620639 | 0 | 401 | 272 | 0.994 |
|  |  |  | 1 | 43 | 27 |  |
|  |  |  | 2 | 0 | 1 |  |
| 14 | NAT12 | rs3825621 | 0 | 352 | 239 | 0.879 |
|  |  |  | 1 | 84 | 56 |  |
|  |  |  | 2 | 8 | 5 |  |
| 15 | C15orf48 | rs1629871 | 0 | 320 | 253 | NA |
|  |  |  | 1 | 0 | 0 |  |
|  |  |  | 2 | 0 | 0 |  |
| 15 | SPESP1 / NOX5 | rs3743091 | 0 | 409 | 265 | 0.067 |
|  |  |  | 1 | 35 | 34 |  |
|  |  |  | 2 | 0 | 1 |  |
| 15 | SPESP1 / NOX5 | rs3743093 | 0 | 170 | 117 | 0.819 |
|  |  |  | 1 | 206 | 133 |  |
|  |  |  | 2 | 66 | 50 |  |
| 15 | SPESP1 / NOX5 | rs12442417 | 0 | 405 | 277 | 0.470 |
|  |  |  | 1 | 37 | 23 |  |
|  |  |  | 2 | 2 | 0 |  |
| 15 | SPESP1 / NOX5 | rs12899318 | 0 | 151 | 94 | 0.531 |
|  |  |  | 1 | 206 | 146 |  |
|  |  |  | 2 | 86 | 60 |  |
| 15 | SPESP1 / NOX5 | rs12907196 | 0 | 149 | 92 | 0.625 |
|  |  |  | 1 | 204 | 147 |  |
|  |  |  | 2 | 90 | 60 |  |
| 16 | CTRB2 / CTRB1 / BCAR1 | rs4737 | 0 | 199 | 176 | 0.134 |
|  |  |  | 1 | 111 | 68 |  |
|  |  |  | 2 | 10 | 9 |  |
| 16 | CTRB2 / CTRB1 / BCAR1 | rs889515 | 0 | 283 | 205 | 0.250 |
|  |  |  | 1 | 146 | 83 |  |
|  |  |  | 2 | 14 | 10 |  |
| 16 | CTRB2 / CTRB1 / BCAR1 | rs8056814 | 0 | 382 | 238 | 0.021 |
|  |  |  | 1 | 58 | 58 |  |
|  |  |  | 2 | 4 | 4 |  |
| 16 | CTRB2 / CTRB1 / BCAR1 | rs13331385 | 0 | 279 | 190 | 0.001 |
|  |  |  | 1 | 39 | 58 |  |
|  |  |  | 2 | 2 | 3 |  |
| 16 | CTRB2 / CTRB1 / BCAR1 | rs12924999 | 0 | 287 | 202 | 0.585 |
|  |  |  | 1 | 142 | 87 |  |
|  |  |  | 2 | 13 | 10 |  |
| 16 | CTRB2 / CTRB1 / BCAR1 | rs5842 | 0 | 97 | 66 | 0.185 |
|  |  |  | 1 | 153 | 121 |  |
|  |  |  | 2 | 70 | 65 |  |
| 16 | CTRB2 / CTRB1 / BCAR1 | rs7190458 | 0 | 395 | 270 | 0.478 |
|  |  |  | 1 | 33 | 29 |  |
|  |  |  | 2 | 1 | 0 |  |
| 16 | CTRB2 / CTRB1 / BCAR1 | rs4887810 | 0 | 222 | 127 | 0.063 |
|  |  |  | 1 | 170 | 139 |  |
|  |  |  | 2 | 43 | 32 |  |
| 17 | METT10D | rs4613098 | 0 | 265 | 171 | 0.611 |
|  |  |  | 1 | 150 | 110 |  |
|  |  |  | 2 | 28 | 18 |  |
| 17 | METT10D | rs4790335 | 0 | 186 | 146 | 0.645 |
|  |  |  | 1 | 220 | 113 |  |
|  |  |  | 2 | 37 | 39 |  |
| 17 | METT10D | rs3744276 | 0 | 251 | 164 | 0.589 |
|  |  |  | 1 | 159 | 110 |  |
|  |  |  | 2 | 32 | 24 |  |
| 17 | METT10D | rs8077068 | 0 | 185 | 148 | 0.404 |
|  |  |  | 1 | 219 | 116 |  |
|  |  |  | 2 | 38 | 36 |  |
| 17 | METT10D | rs2078261 | 0 | 325 | 225 | 0.619 |
|  |  |  | 1 | 110 | 69 |  |
|  |  |  | 2 | 9 | 6 |  |
| 17 | METT10D | rs7215857 | 0 | 325 | 226 | 0.547 |
|  |  |  | 1 | 108 | 68 |  |
|  |  |  | 2 | 10 | 6 |  |
| 17 | METT10D | rs11871981 | 0 | 364 | 245 | 0.545 |
|  |  |  | 1 | 77 | 49 |  |
|  |  |  | 2 | 1 | 5 |  |
| 17 | METT10D | rs11871188 | 0 | 326 | 227 | 0.620 |
|  |  |  | 1 | 106 | 66 |  |
|  |  |  | 2 | 10 | 7 |  |
| 17 | METT10D | rs4268798 | 0 | 138 | 87 | 0.470 |
|  |  |  | 1 | 217 | 147 |  |
|  |  |  | 2 | 87 | 64 |  |
| 17 | METT10D | rs9892878 | 0 | 244 | 173 | 0.773 |
|  |  |  | 1 | 169 | 103 |  |
|  |  |  | 2 | 30 | 24 |  |
| 17 | METT10D | rs2028600 | 0 | 242 | 172 | 0.728 |
|  |  |  | 1 | 171 | 103 |  |
|  |  |  | 2 | 30 | 24 |  |
| 17 | METT10D | rs7207104 | 0 | 166 | 116 | 0.782 |
|  |  |  | 1 | 209 | 130 |  |
|  |  |  | 2 | 68 | 54 |  |
| 17 | METT10D | rs7213126 | 0 | 344 | 234 | 0.728 |
|  |  |  | 1 | 91 | 62 |  |
|  |  |  | 2 | 9 | 4 |  |
| 17 | METT10D | rs9303083 | 0 | 347 | 235 | 0.793 |
|  |  |  | 1 | 89 | 60 |  |
|  |  |  | 2 | 8 | 4 |  |
| 17 | METT10D | rs935134 | 0 | 335 | 218 | 0.418 |
|  |  |  | 1 | 95 | 78 |  |
|  |  |  | 2 | 9 | 4 |  |
| 17 | METT10D | rs8078195 | 0 | 369 | 235 | 0.036 |
|  |  |  | 1 | 70 | 58 |  |
|  |  |  | 2 | 3 | 7 |  |
| 17 | METT10D | rs11078210 | 0 | 249 | 161 | 0.262 |
|  |  |  | 1 | 170 | 116 |  |
|  |  |  | 2 | 23 | 23 |  |
| 18 | DCC | rs7506328 | 0 | 152 | 111 | 0.630 |
|  |  |  | 1 | 214 | 136 |  |
|  |  |  | 2 | 76 | 52 |  |
| 18 | DCC | rs6417121 | 0 | 105 | 77 | 0.653 |
|  |  |  | 1 | 231 | 152 |  |
|  |  |  | 2 | 105 | 70 |  |
| 18 | DCC | rs8089704 | 0 | 297 | 189 | 0.744 |
|  |  |  | 1 | 121 | 102 |  |
|  |  |  | 2 | 25 | 9 |  |
| 18 | DCC | rs9954344 | 0 | 181 | 135 | 0.541 |
|  |  |  | 1 | 203 | 120 |  |
|  |  |  | 2 | 60 | 44 |  |
| 18 | DCC | rs9950970 | 0 | 181 | 133 | 0.651 |
|  |  |  | 1 | 202 | 123 |  |
|  |  |  | 2 | 60 | 44 |  |
| 18 | DCC | rs1367634 | 0 | 178 | 132 | 0.599 |
|  |  |  | 1 | 201 | 124 |  |
|  |  |  | 2 | 61 | 44 |  |
| 18 | DCC | rs12954274 | 0 | 180 | 133 | 0.611 |
|  |  |  | 1 | 199 | 121 |  |
|  |  |  | 2 | 62 | 45 |  |
| 18 | DCC | rs12969525 | 0 | 363 | 241 | 0.828 |
|  |  |  | 1 | 73 | 57 |  |
|  |  |  | 2 | 7 | 2 |  |
| 18 | DCC | rs11873306 | 0 | 366 | 243 | 0.653 |
|  |  |  | 1 | 69 | 52 |  |
|  |  |  | 2 | 8 | 5 |  |
| 18 | DCC | rs2339639 | 0 | 236 | 164 | 0.668 |
|  |  |  | 1 | 174 | 115 |  |
|  |  |  | 2 | 32 | 20 |  |
| 18 | DCC | rs2270951 | 0 | 122 | 94 | 0.577 |
|  |  |  | 1 | 232 | 142 |  |
|  |  |  | 2 | 89 | 63 |  |
| 18 | DCC | rs9807370 | 0 | 123 | 94 | 0.607 |
|  |  |  | 1 | 231 | 142 |  |
|  |  |  | 2 | 89 | 63 |  |
| 18 | DCC | rs17507725 | 0 | 123 | 93 | 0.603 |
|  |  |  | 1 | 227 | 140 |  |
|  |  |  | 2 | 92 | 64 |  |
| 18 | DCC | rs8095329 | 0 | 314 | 218 | 0.614 |
|  |  |  | 1 | 123 | 77 |  |
|  |  |  | 2 | 7 | 5 |  |
| 18 | DCC | rs869224 | 0 | 134 | 103 | 0.747 |
|  |  |  | 1 | 225 | 133 |  |
|  |  |  | 2 | 81 | 62 |  |
| 18 | DCC | rs17417046 | 0 | 130 | 101 | 0.581 |
|  |  |  | 1 | 218 | 131 |  |
|  |  |  | 2 | 93 | 67 |  |
| 18 | C18orf55 / FBXO15 | rs17088751 | 0 | 369 | 246 | 0.777 |
|  |  |  | 1 | 65 | 52 |  |
|  |  |  | 2 | 8 | 0 |  |
| 18 | C18orf55 / FBXO15 | rs4891534 | 0 | 292 | 195 | 0.944 |
|  |  |  | 1 | 136 | 99 |  |
|  |  |  | 2 | 13 | 5 |  |
| 18 | C18orf55 / FBXO15 | rs12955042 | 0 | 349 | 230 | 0.575 |
|  |  |  | 1 | 90 | 65 |  |
|  |  |  | 2 | 5 | 4 |  |
| 18 | C18orf55 / FBXO15 | rs7228051 | 0 | 238 | 136 | 0.101 |
|  |  |  | 1 | 165 | 135 |  |
|  |  |  | 2 | 39 | 26 |  |
| 18 | C18orf55 / FBXO15 | rs8086078 | 0 | 405 | 275 | 0.831 |
|  |  |  | 1 | 39 | 25 |  |
|  |  |  | 2 | 0 | 0 |  |
| 18 | C18orf55 / FBXO15 | rs9949477 | 0 | 334 | 224 | 0.806 |
|  |  |  | 1 | 95 | 72 |  |
|  |  |  | 2 | 12 | 3 |  |
| 18 | C18orf55 / FBXO15 | rs3813119 | 0 | 253 | 195 | 0.605 |
|  |  |  | 1 | 58 | 51 |  |
|  |  |  | 2 | 4 | 3 |  |
| 18 | C18orf55 / FBXO15 | rs3813114 | 0 | 279 | 219 | 0.989 |
|  |  |  | 1 | 37 | 34 |  |
|  |  |  | 2 | 3 | 0 |  |
| 18 | C18orf55 / FBXO15 | rs3813108 | 0 | 408 | 274 | 0.902 |
|  |  |  | 1 | 36 | 25 |  |
|  |  |  | 2 | 0 | 0 |  |
| 18 | C18orf55 / FBXO15 | rs3737512 | 0 | 403 | 273 | 0.811 |
|  |  |  | 1 | 36 | 26 |  |
|  |  |  | 2 | 0 | 0 |  |
| 18 | C18orf55 / FBXO15 | rs17088882 | 0 | 383 | 255 | 0.839 |
|  |  |  | 1 | 54 | 43 |  |
|  |  |  | 2 | 5 | 1 |  |
| 19 | NDUFS7 / GAMT / DAZAP1 | rs3848638 | 0 | 131 | 85 | 0.670 |
|  |  |  | 1 | 214 | 147 |  |
|  |  |  | 2 | 96 | 68 |  |
| 19 | NDUFS7 / GAMT / DAZAP1 | rs11666067 | 0 | 179 | 117 | 0.475 |
|  |  |  | 1 | 197 | 131 |  |
|  |  |  | 2 | 65 | 51 |  |
| 19 | NDUFS7 / GAMT / DAZAP1 | rs7251282 | 0 | 319 | 252 | 0.867 |
|  |  |  | 1 | 1 | 1 |  |
|  |  |  | 2 | 0 | 0 |  |
| 19 | NDUFS7 / GAMT / DAZAP1 | rs11666805 | 0 | 380 | 254 | 0.850 |
|  |  |  | 1 | 52 | 38 |  |
|  |  |  | 2 | 4 | 2 |  |
| 19 | C3 / GPR108 | rs11569536 | 0 | 369 | 270 | 0.006 |
|  |  |  | 1 | 70 | 29 |  |
|  |  |  | 2 | 3 | 0 |  |
| 19 | C3 / GPR108 | rs344542 | 0 | 175 | 115 | 0.909 |
|  |  |  | 1 | 199 | 138 |  |
|  |  |  | 2 | 64 | 42 |  |
| 19 | C3 / GPR108 | rs3745565 | 0 | 365 | 227 | 0.015 |
|  |  |  | 1 | 74 | 64 |  |
|  |  |  | 2 | 5 | 9 |  |
| 19 | C3 / GPR108 | rs423490 | 0 | 252 | 190 | 0.079 |
|  |  |  | 1 | 157 | 94 |  |
|  |  |  | 2 | 32 | 16 |  |
| 19 | C3 / GPR108 | rs428453 | 0 | 198 | 126 | 0.499 |
|  |  |  | 1 | 187 | 131 |  |
|  |  |  | 2 | 58 | 42 |  |
| 19 | C3 / GPR108 | rs2230205 | 0 | 338 | 226 | 0.502 |
|  |  |  | 1 | 98 | 67 |  |
|  |  |  | 2 | 4 | 6 |  |
| 19 | C3 / GPR108 | rs1047286 | 0 | 243 | 209 | 8.22E-05 |
|  |  |  | 1 | 159 | 76 |  |
|  |  |  | 2 | 38 | 14 |  |
| 19 | C3 / GPR108 | rs2230199 | 0 | 236 | 205 | 3.71E-05 |
|  |  |  | 1 | 167 | 80 |  |
|  |  |  | 2 | 40 | 14 |  |
| 19 | C3 / GPR108 | rs339392 | 0 | 286 | 186 | 0.177 |
|  |  |  | 1 | 143 | 96 |  |
|  |  |  | 2 | 13 | 18 |  |
| 19 | C3 / GPR108 | rs339400 | 0 | 352 | 227 | 0.050 |
|  |  |  | 1 | 84 | 64 |  |
|  |  |  | 2 | 2 | 8 |  |
| 20 | C20orf19 | rs6133075 | 0 | 406 | 272 | 0.614 |
|  |  |  | 1 | 36 | 23 |  |
|  |  |  | 2 | 1 | 3 |  |
| 20 | C20orf19 | rs6137194 | 0 | 189 | 128 | 0.804 |
|  |  |  | 1 | 208 | 144 |  |
|  |  |  | 2 | 45 | 27 |  |
| 20 | TOMM34 | rs1804644 | 0 | 421 | 279 | 0.254 |
|  |  |  | 1 | 21 | 20 |  |
|  |  |  | 2 | 1 | 1 |  |
| 20 | TOMM34 | rs6103938 | 0 | 176 | 129 | 0.189 |
|  |  |  | 1 | 203 | 139 |  |
|  |  |  | 2 | 61 | 31 |  |

^a^ This SNP failed using an Illumina 384 SNP platform. These results were obtained upon re-genotyping of the SNP using TaqMan.
