# Supplementary material for: Evaluation of Clustering and Genotype Distribution for Replication in Genome Wide Association Studies: The Age-Related Eye Disease Study
Source: PLoS One. 2008 Nov 26;3(11):e3813. doi: 10.1371/journal.pone.0003813 (PMC2583911; doi:10.1371/journal.pone.0003813)
Supplement: Table S4 — Genotype distribution results for 6 SNPs with significant log-additive p-values in Mayo samples compared to genotype distribution results for the same SNPs genotyped in AREDS samples (replication cohort). (0.06 MB DOC) [file pone.0003813.s004.doc]

|  |  |  |  | **DISCOVERY COHORT (MAYO)** | | |  | **REPLICATION COHORT (AREDS)** | | |
| --- | --- | --- | --- | --- | --- | --- | --- | --- | --- | --- |
| **Chromosome** | **AREDS Locus** | **SNP** | **Genotype (# minor alleles)** | **AMD** | **Controls** | **Additive p-value** |  | **AMD** | **Controls** | **Additive p-value** |
| 1 | LOC127602 | rs1871570 | 0 | 113 | 99 | 0.009 |  | 361 | 89 | 0.49 |
|  |  |  | 1 | 225 | 155 |  |  | 629 | 159 |  |
|  |  |  | 2 | 104 | 46 |  |  | 259 | 55 |  |
| 1 | LOC127602 | rs12038394 | 0 | 159 | 85 | 0.02 |  | 442 | 112 | 0.7 |
|  |  |  | 1 | 214 | 157 |  |  | 613 | 149 |  |
|  |  |  | 2 | 68 | 58 |  |  | 203 | 48 |  |
| 7 | NOD1 | rs2906766 | 0 | 240 | 126 | 0.009 |  | 599 | 139 | 0.38 |
|  |  |  | 1 | 158 | 140 |  |  | 532 | 130 |  |
|  |  |  | 2 | 43 | 32 |  |  | 134 | 41 |  |
| 7 | PLXNA4B | rs2341823 | 0 | 365 | 262 | 0.04 |  | 1054 | 247 | 0.01 |
|  |  |  | 1 | 73 | 35 |  |  | 193 | 52 |  |
|  |  |  | 2 | 4 | 1 |  |  | 16 | 13 |  |
| 7 | PLXNA4B | rs1499300 | 0 | 296 | 226 | 0.01 |  | 883 | 209 | 0.77 |
|  |  |  | 1 | 135 | 72 |  |  | 350 | 98 |  |
|  |  |  | 2 | 11 | 2 |  |  | 35 | 4 |  |
| 7 | PLXNA4B | rs11773117 | 0 | 318 | 235 | 0.01 |  | 943 | 240 | 0.34 |
|  |  |  | 1 | 113 | 65 |  |  | 299 | 71 |  |
|  |  |  | 2 | 12 | 0 |  |  | 24 | 3 |  |
